# PD-L1 up-regulation in melanoma increases disease aggressiveness and is mediated through miR-17-5p

**DOI:** 10.18632/oncotarget.15213

**Published:** 2017-02-09

**Authors:** Valentina Audrito, Sara Serra, Aureliano Stingi, Francesca Orso, Federica Gaudino, Cinzia Bologna, Francesco Neri, Giulia Garaffo, Romina Nassini, Gianna Baroni, Eliana Rulli, Daniela Massi, Salvatore Oliviero, Roberto Piva, Daniela Taverna, Mario Mandalà, Silvia Deaglio

**Affiliations:** ^1^ Human Genetics Foundation (HuGeF), Turin, Italy; ^2^ Department of Medical Sciences, University of Turin, Turin, Italy; ^3^ Department of Molecular Biotechnology and Health Sciences, University of Turin, Turin, Italy; ^4^ Department of Health Sciences, University of Florence, Italy; ^5^ Department of Surgery and Translational Medicine, University of Florence, Italy; ^6^ Methodology for Clinical Research Laboratory, IRCCS – Istituto di Ricerche Farmacologiche Mario Negri, Milan, Italy; ^7^ Department of Life Sciences and Systems Biology, University of Turin, Turin, Italy; ^8^ Department of Oncology and Hematology, Papa Giovanni XXIII Hospital, Bergamo, Italy

**Keywords:** melanoma, targeted therapy, resistance to therapy, microRNA, regulation of gene expression

## Abstract

PD-L1 is expressed by a subset of patients with metastatic melanoma (MM) with an unfavorable outcome. Its expression is increased in cells resistant to BRAF or MEK inhibitors (BRAFi or MEKi). However, the function and regulation of expression of PD-L1 remain incompletely understood.

After generating BRAFi- and MEKi-resistant cell lines, we observed marked up-regulation of PD-L1 expression. These cells were characterized by a common gene expression profile with up-regulation of genes involved in cell movement. Consistently, *in vitro* they showed significantly increased invasive properties. This phenotype was controlled in part by PD-L1, as determined after silencing the molecule. Up-regulation of PD-L1 was due to post-transcriptional events controlled by miR-17-5p, which showed an inverse correlation with PD-L1 mRNA. Direct binding between miR-17-5p and the 3’-UTR of PD-L1 mRNA was demonstrated using luciferase reporter assays.

In a cohort of 80 BRAF-mutated MM patients treated with BRAFi or MEKi, constitutive expression of PD-L1 in the absence of immune infiltrate, defined the patient subset with the worst prognosis. Furthermore, PD-L1 expression increased in tissue biopsies after the metastatic lesions became resistant to BRAFi or MEKi. Lastly, plasmatic miR-17-5p levels were higher in patients with PD-L1^+^ than PD-L1^-^ lesions.

In conclusion, our findings indicate that PD-L1 expression induces a more aggressive behavior in melanoma cells. We also show that PD-L1 up-regulation in BRAFi or MEKi-resistant cells is partly due to post-transcriptional mechanisms that involve miR-17-5p, suggesting that miR-17-5p may be used as a marker of PD-L1 expression by metastatic lesions and ultimately a predictor of responses to BRAFi or MEKi.

## INTRODUCTION

The therapy of metastatic melanoma (MM) was radically changed by the introduction of inhibitors of the BRAF oncogene, which is mutated in ≈40-50% of patients. The BRAF inhibitors (BRAFi) vemurafenib and dabrafenib were proved to be more successful than conventional chemotherapy in the treatment of these patients in terms of activity and efficacy, achieving partial and complete remissions in many instances [1, 2]. However, resistance to BRAFi typically emerges a few months after beginning of therapy [3]. Following prolonged treatment with BRAFi, some patients also develop secondary tumors [4]. Both these phenomena are attributed to the paradoxical activation of MEK/ERK signaling consequent to the upstream block induced by BRAFi [5]. This finding was the rationale for the introduction of MEK inhibitors (MEKi) in the management of these patients [6]. The combination of BRAFi with MEKi was proposed as a strategy to delay or even prevent the onset of resistance, without increasing the risk of developing secondary cancers. Three large, prospective, randomized clinical trials indicate that combined therapy is significantly more effective than either drug used alone and that resistance occurs at a later stage, proposing this combination as the new standard treatment for this subset of MM patients [7–9].

The alternative or complementary therapeutic strategy for patients with MM is to restore immune functions, boosting T cell specific responses against the tumor [10]. Among the immune checkpoint targets of clinical importance is PD-1, which is expressed by exhausted T lymphocytes [11]. PD-1 binds to the PD-L1 ligand, which may be expressed by tumor cells, including melanoma [12, 13]. Recent clinical trials with anti-PD-1 antibodies (nivolumab and pembrolizumab) have demonstrated higher objective response rates and increased overall survival compared to chemotherapy [14–16], albeit at the cost of significant immune-related toxicities, particularly when used in combination with anti-CTLA-4 antibodies [17, 18].

Regulation of PD-L1 expression by melanoma is an area of intense investigation. On the one side, PD-L1 expression is induced by interferon-gamma, in turn produced by activated CD8^+^ T lymphocytes, highlighting immune escape mechanisms [19–22]. On the other side, PD-L1 may be induced after paradoxical activation of the MAP kinases, as recently shown [23, 24]. Furthermore, PTEN loss was found to up-regulate PD-L1, likely through the over-activation of the PI3K/Akt pathway, at least in other tumor models [25, 26].

In our previous work, we showed that out of a panel of 12 melanoma cell lines, A375 was the only one carrying mutations in the *BRAF* oncogene where a distinct population of PD-L1^+^ cells could be defined. The sorted PD-L1^+^ subset of the A375 cell line was characterized by a highly invasive phenotype, with an enhanced ability to grow in xenograft models. This phenotype was attributed to the transcriptional modulation of a set of genes involved in adhesion and migration [27].

In the present work we directly link expression of PD-L1 to a more aggressive behavior of melanoma cell lines. This finding is substantiated by data obtained in patients, where intrinsic PD-L1 expression defines a subset of patients with the most unfavorable prognosis. Furthermore, we define a novel post-transcriptional circuit responsible for PD-L1 up-regulation in BRAFi-resistant melanoma cells, which is based on the direct interaction between the 3’-UTR mRNA of PD-L1 and miR-17-5p. Lastly, we show that miR-17-5p levels in patients with metastatic melanoma inversely correlate with PD-L1 expression and may predict sensitivity to BRAFi.

## RESULTS

### Resistance to BRAFi and MEKi is accompanied by induction of PD-L1 expression in BRAF^V600E^-mutated melanoma cell lines

**Figure 1:**
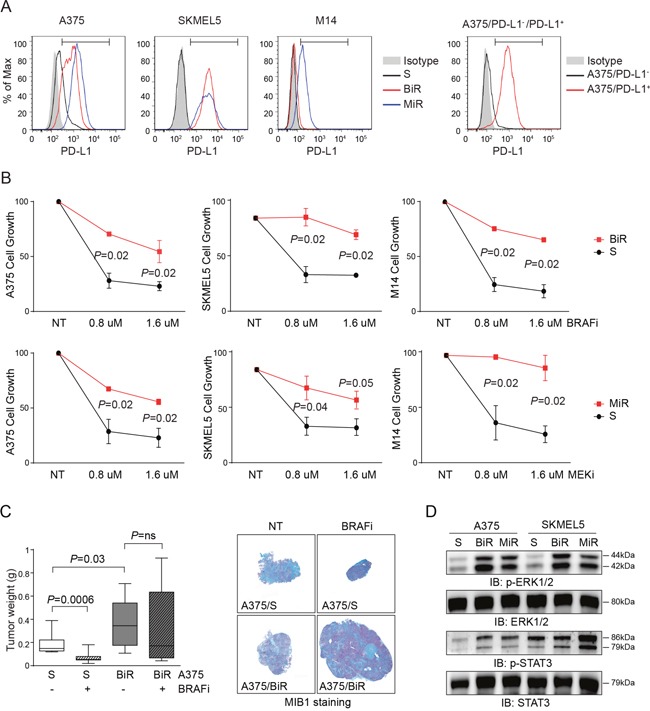
Establishment and characterization of BRAFV600E-mutated melanoma cell lines resistant to BRAFi and MEKi **A**. Flow-cytometric histogram plots reporting surface expression of PD-L1 by A375, A375/PD-L1^+^, SKMEL5, M14 cell lines, before (S cells) and after the acquisition of resistance to BRAFi (BiR) and MEKi (MiR). The bars within the histogram plot define the positive cut-off. **B**. Cell growth of A375, SKMEL5 and M14 melanoma cell lines sensitive (black line) or resistant (red line) to BRAFi (Dabrafenib) or MEKi (Pimasertib) used at scalar doses for 72 hours as measured by MTT assay. Data are represented as % of control (untreated cells). Data from 3 independent experiments, each performed in triplicate. **C**. Box plot showing tumor weight (g) of A375/S or /BiR cells after subcutaneous injection of 10^7^ cells in matrigel in NOD/SCID mice (n=5). Cells were left to grow for 14 days before beginning treatment with BRAFi (30 mg/kg/daily gavage) for 1 week. Mice were then sacrificed and lesions stained for MIB1 to determine the proliferative fraction (representative images are shown). Original magnification x2.5 (left panels), scale bar 50 μm. **D**. Western blot analysis of p-ERK1/2 and pSTAT3, and the corresponding total protein of A375 and SKMEL5 S, BiR and MiR. S: sensitive, BiR: BRAFi resistant, MiR: MEKi resistant.

The BRAFV600E mutated A375 (20% of cells constitutively expressing PD-L1), SKMEL5 and M14 (both PD-L1^-^, Figure 1A) cell lines were rendered resistant to BRAFi or MEKi by repeated exposure to increasing concentrations of each drug. Resistant cells are indicated as BiR and MiR, respectively. Doses were slowly escalated over a period of 12 weeks to reach a plateau of 1.6 μM for both drugs. Resistance to BRAFi or MEKi was confirmed using the MTT assay (Figure 1B), as well as in xenograft models where A375/BiR, the cell line selected for *in vivo* experiments, failed to respond to treatment with dabrafenib, at variance with control cells (Figure 1C). No double-resistant cell line could be stabilized, at least under these experimental conditions. In these cell lines, resistance to BRAFi and MEKi was accompanied by paradoxical activation of ERK1/2 tyrosine kinase and STAT3 downstream activation (Figure 1D).

**Figure 2:**
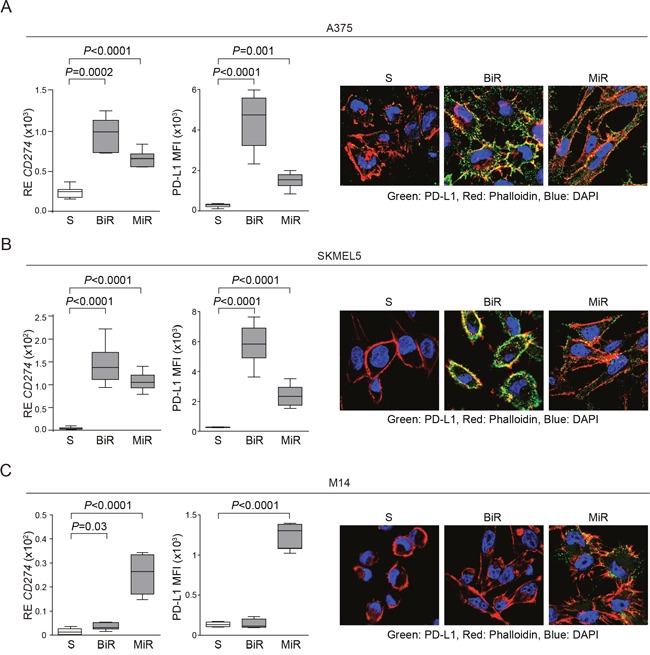
BRAF or MEK inhibitors resistance is accompanied by PD-L1 overexpression **A-C**. Expression of *CD274*/PD-L1 at the mRNA (left graph) and protein levels, analyzed as Mean Fluorescence Intensity (MFI, middle graph) in A375 **A**., SKMEL5 **B**. and M14 **C**. melanoma cell lines. Surface expression was confirmed by confocal microscopy analysis. Original magnification x63.

MiR cell lines were characterized by a robust up-modulation of *CD274*/PD-L1, both at the mRNA and protein levels (Figure 2A-2C). Among BiR lines, expression of PD-L1 increased in A375 (from 20% to 100%) and SKMEL5 (from 0% to 100%), while M14 remained PD-L1^-^ (Figure 2A-2C). Confocal microscopy and flow cytometry analyses confirmed that PD-L1 was intensely expressed at the cell surface (Figure 2A-2C and Figure 1A).

### BRAFi- and MEKi-resistant cell lines show a distinct gene profile, which partially overlaps with that of A375/PD-L1^+^ cells

**Figure 3:**
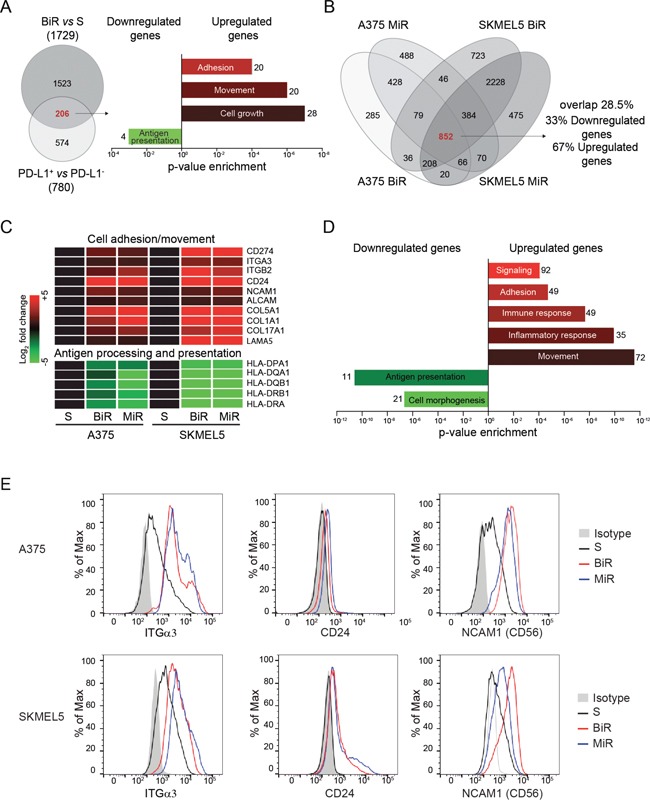
PD-L1+ BRAFi or MEKi-resistant melanoma cells are characterized by a distinct genetic profile **A**. Venn diagram showing genes commonly modulated when comparing A375/BiR cells to the A375/PD-L1^+^ clone. The histograms represent enriched gene categories obtained after analyzing the 206 common genes. **B**. Venn diagrams showing 852 common differentially expressed genes in BiR and MiR A375 and SKMEL5 cell lines obtained by RNAseq analysis. **C**. Most representative up-regulated (red) and down-regulated (green) differentially expressed genes belong to the common 852 genes. **D**. Histograms represent the more enriched GO categories in the resistant variants. **E**. Differential expression of integrin-α3 (ITGα3), CD24 and NCAM1 (CD56) protein of sensitive and BRAFi or MEKi-resistant melanoma cells by FACS analysis.

We previously reported that the PD-L1^+^ variant of the A375 cell line is characterized by a specific gene expression profile [27]. We now compared the genetic signature of A375/BiR to that of A375/PD-L1^+^ cells and found 206 commonly modulated genes (Figure 3A and Supplementary Table 1). Among them, the most significantly up-modulated genes pertained to the “adhesion”, “movement” and “cell growth” categories, while “antigen presentation” was the most significantly down-modulated gene category. This signature suggests that cell movement and immune escape are mechanisms shared between the A375/PD-L1^+^ and the A375/BiR variants (Figure 3A). We then compared the RNA sequencing profiles of A375/BiR and /MiR and those of SKMEL5/BiR and /MiR and identified 852 genes that were similarly modulated in the four line variants (Supplementary Table 2). Among these genes, 574 (67%) were up-regulated and 278 (33%) were down-regulated (Figure 3B). *CD274* (PD-L1) was among the most significantly overexpressed genes in the BiR and MiR variants, confirming the validity of the approach (Figure 3C). Gene ontology (GO) analysis confirmed that the main biological processes modulated during the acquisition of resistance concerned cell movement and immune responses (Figure 3D). Specifically, genes involved in cell adhesion, movement, signaling and immune/inflammatory responses were significantly up-regulated, while genes involved in antigen processing and presentation and cell morphogenesis were down-regulated (Figure 3C-3D). Among these genes, we confirmed up-regulation of integrin α3 (ITGα3), CD24 and NCAM1 (CD56) at the protein level (Figure 3E), which have been previously connected to increased aggressiveness of melanoma cells. Specifically, ITGα3 is expressed by melanoma cells with a highly invasive potential [28], CD24 is considered a negative prognostic factor for patients with cutaneous melanoma [29], while CD56 is a neural marker, which can be expressed by melanomas with desmoplastic and spindle cell differentiation [30].

### Over-expression of PD-L1 in BRAFi- and MEKi-resistant cell lines contributes to increased invasiveness

**Figure 4:**
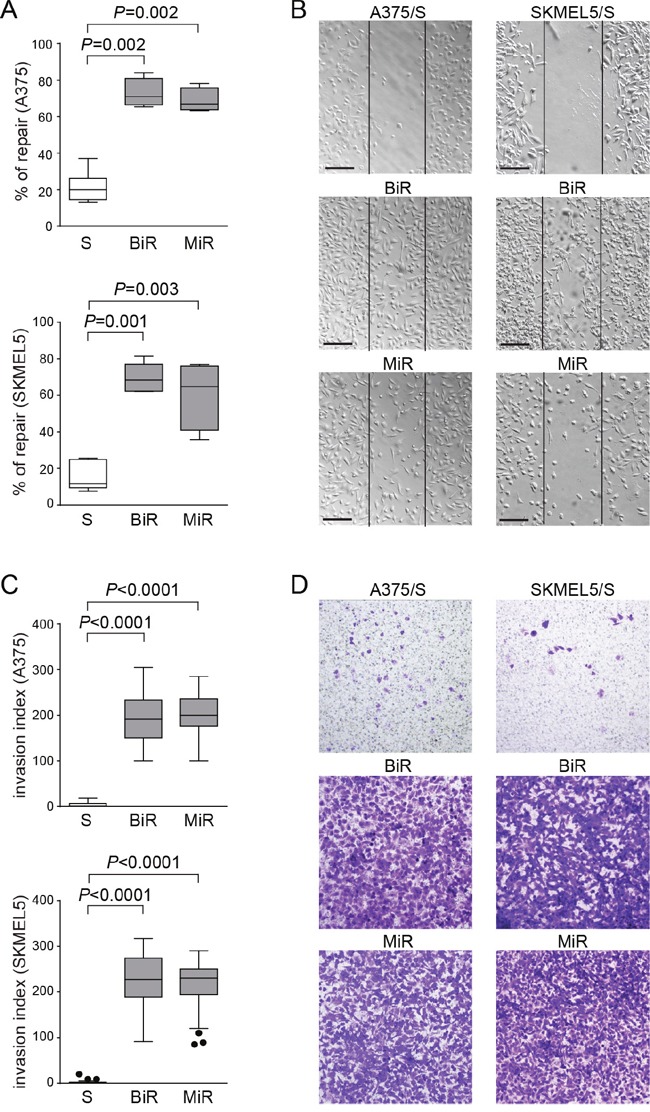
Over-expression of PD-L1 in BRAFi- and MEKi-resistant cell lines contributes to increased invasiveness **A**. Box plots showing % of repair in A375 and SKMEL5 variants, determined by measuring wound area ratio between two time points at 24 hours and t=0. **B**. Representative pictures (x10 magnification, scale bar 200 μm) of wound-healing assay comparing S, BiR and MiR variants. **C**. Box plots representing cumulative data of invasion assays performed using A375 and SKMEL5 cell variants. **D**. Representative images (x10 magnification) of A375 (left panels) and SKMEL5 (right panels) cell lines comparing S, BiR and MiR variants.

Following the gene expression profiles, we hypothesized that BiR and MiR cells would show a more aggressive behavior when compared to the drug-sensitive counterparts. Increased motility and aggressiveness of A375/PD-L1^+^ cells compared to A375/PD-L1^-^ cells was previously confirmed both *in vitro* and *in vivo* [27]. We now extended these studies to A375 and SKMEL5 /BiR and /MiR, by analyzing their ability to repair wounds. Wound-healing assays performed at 48 hours clearly demonstrated that both /BiR and /MiR A375 and SKMEL5 cells repaired the wound in a more efficient way than the sensitive (S) counterpart (Figure 4A-4B). A375 cells showed a mean % of repair of 21±2.5% *vs* 73±4% of A375/BiR *vs* 69±3% of A375/MiR (Figure 4A-4B). SKMEL5 cells showed a mean of % of repair of 15±2.5% *vs* 69±4% of SKMEL5/BiR *vs* 60±9% of SKMEL5/MiR (Figure 4A-4B). Likewise BiR and MiR cells showed significantly increased chemotactic and invasive performances as compared to the S counterparts (Figure 4C-4D and Supplementary Figure 1). Invasion index for A375 was 3.5±5 SD *vs* 193±51 SD of A375/BiR and 197±48 SD of A375/MiR. Invasion index for SKMEL5 was 1.7±3 SD *vs* 226±53 SD for SKMEL5/BiR and 213±50 SD for SKMEL5/MiR (Figure 4C).

These results indicate that cells that constitutively express PD-L1 and cells that acquire PD-L1 as a consequence of the BiR state show modulation of common genes and acquire similar behavioral patterns.

### Silencing of PD-L1 expression influences aggressiveness

**Figure 5:**
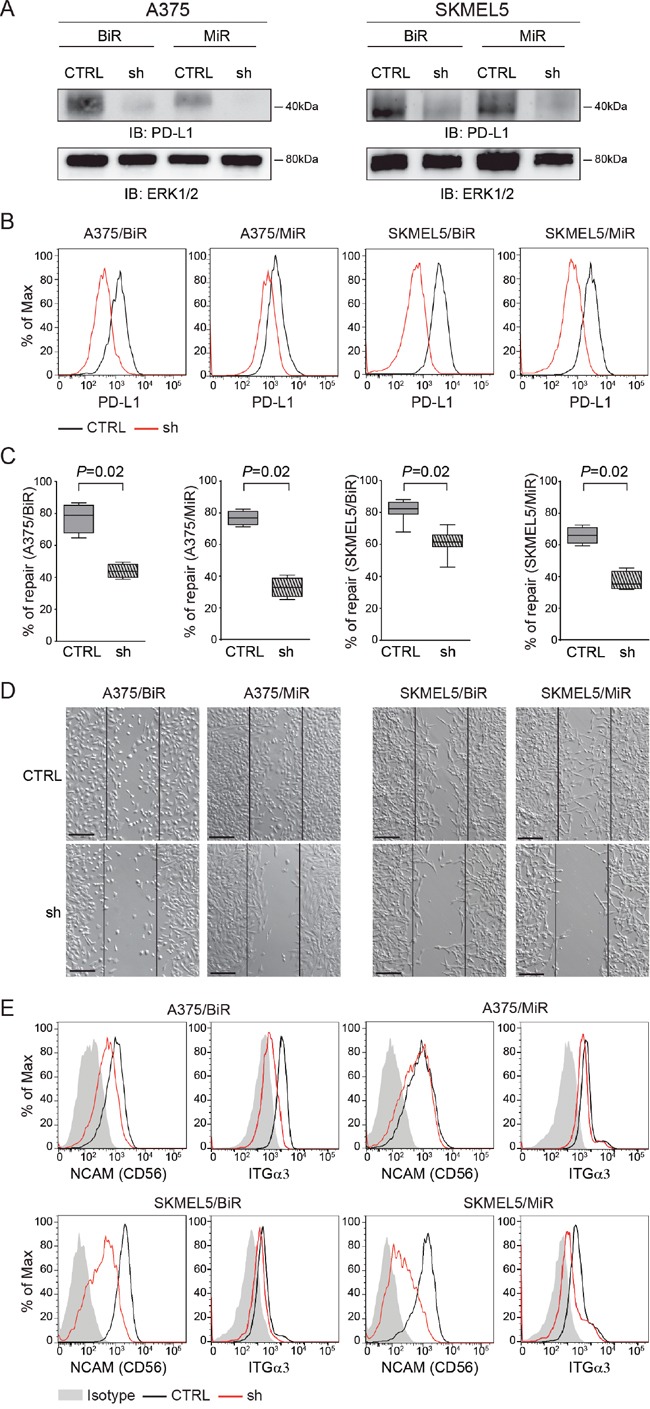
PD-L1 silencing influences aggressiveness **A**. PD-L1 protein level of silenced infected cells (sh) in comparison to the infected control (CTRL) in A375/BiR, A375/MiR, SKMEL5/BiR and SKMEL5/MiR cells. **B**. Flow-cytometric histogram plots of PD-L1-silenced A375 and SKMEL5 resistant cell lines. **C**. Box plot of wound healing repair of A375/BiR, A375/MiR, SKMEL5/BiR, SKMEL5/MiR CTRL *vs* sh. **D**. Representative pictures (x10 magnification, scale bar 200 μm) of wound-healing assay comparing the infected control (CTRL) versus the infected (sh) in A375/BiR, A375/MiR and SKMEL5/BiR and SKMEL5/MiR cells. **E**. Histogram representing CD56 and ITGα3 surface protein expression in CTRL and sh A375/BiR and SKMEL5/BiR cells.

We then asked whether PD-L1 was directly involved in determining this phenotype. To answer this question, A375/BiR or /MiR and SKMEL5/BiR or /MiR cells were infected with a lentivirus carrying a PD-L1-specific (sh) or a control shRNA (CTLR). Cells were then repeatedly selected by cloning and sorting resulting in different clones with a marked decrease in PD-L1 surface levels, as determined by flow cytometry and western blot analysis in all cell line variants (Figure 5A-5B). Silencing of PD-L1 significantly decreased the ability of A375/BiR and /MiR and of SKMEL5/BiR and /MiR cells to repair a wound (Figure 5C-5D). A375/BiR CTRL cells showed a mean % of repair of 77±9% *vs* 44±4% of A375/BiR sh, A375/MiR CTRL 76±6% *vs* 32±6% of A375/MiR sh (Figure 5C-5D). SKMEL5/BiR CTRL cells showed a mean of % of repair of 83±9% *vs* 62±11% of SKMEL5/BiR sh, SKMEL5/MiR CTRL 66±6% *vs* 37±6% of SKMEL5/MiR sh (Figure 5C-5D). Furthermore, silencing of PD-L1 in resistant cells markedly reduced the expression of CD56, and – to a lesser extent – of ITGα3 (Figure 5E), linking PD-L1 expression to the activation of a genetic program dictating a more aggressive phenotype, characterized by increased motility and invasion.

### miR-17-5p post-transcriptionally regulates PD-L1 expression

**Figure 6:**
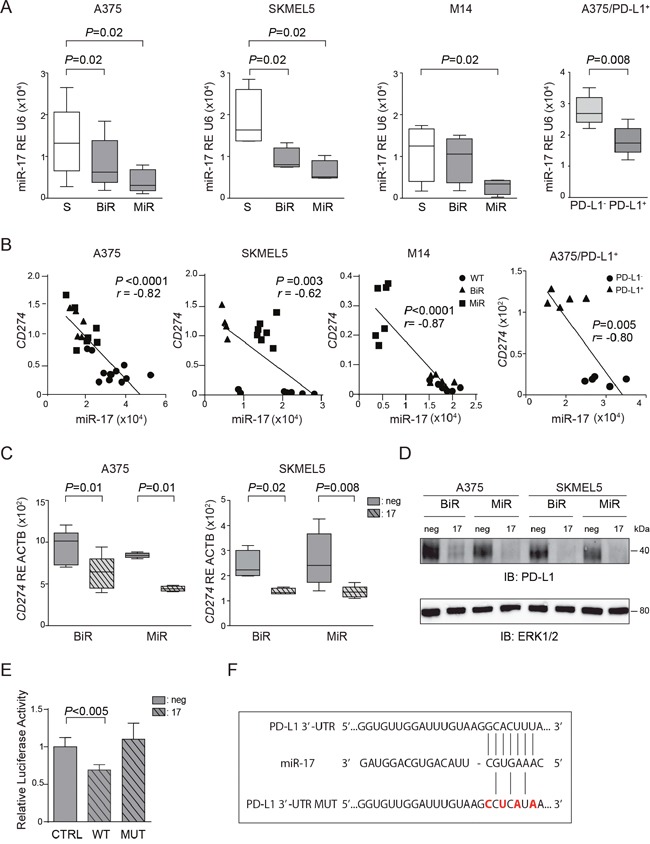
miR-17-5p regulates *CD274*/PD-L1 expression **A**. miR-17-5p basal expression in the sensitive (S) and BiR or MiR melanoma cells in A375, SKMEL5, M14 and A375/PD-L1^+^. **B**. Pearson's correlation of miR-17-5p and PD-L1 level expression in the sensitive (S) and BiR or MiR melanoma cells in A375, SKMEL5, M14 and A375/PD-L1+. **C**. Relative expression of *CD274*/PD-L1 in miR-17-5p transfected cells in comparison to miR-negative transfected control in A375 and SKMEL5 cell line variants. **D**. Representative blot for PD-L1 protein of BiR or MiR of A375 and SKMEL5 transfected cells with pre-miR-17-5p (indicated as 17) and negative control (indicated as neg). **E**. Relative luciferase activity of 293T cells transfected with miR-17-5p (indicated as 17) or negative control (indicated as neg) together with the luciferase vector containing PD-L1 3’-UTR wild type (WT) or mutagenized (MUT). **F**. Seeding sites of miR-17-5p in PD-L1 3’-UTR wild type and mutagenized (MUT).

These findings underline the importance of understanding the molecular mechanisms behind PD-L1 up-regulation in BiR or MiR melanoma cell lines. Previous investigators showed that PD-L1 up-regulation is dependent upon the activation of the JAK/STAT signaling pathways, in turn controlled by the MAPK pathway [23]. Consistently, BiR and MiR cell lines showed significant up-regulation of these signaling pathways (Figure 1D). However, exposure to the specific STAT3 inhibitor caused only a moderate decrease in PD-L1 expression, suggesting that other mechanisms are causing its up-regulation in resistant cells [23]. For this reason, we asked whether PD-L1 up-regulation could be attributed, at least partially, to post-transcriptional mechanisms, such as those regulated by microRNAs. Based on computer predictions (TargetScan 6.0) three microRNAs, namely miR-17-5p, miR-155-5p and miR-425-5p were the only three miRs potentially targeting *CD274*/PD-L1 mRNA. However, only miR-17-5p showed an inverse correlation with *CD274*/PD-L1 mRNA levels in A375, SKMEL5 and M14 cell lines. Specifically, miR-17-5p was down-modulated in A375 and SKMEL5 /BiR and /MiR variants, as well as in M14/MiR cells (Figure 6A). No significant modulation of miR-17-5p was observed in M14/BiR cells, where PD-L1 expression was unaffected (Figure 6A). Consistently, the A375/PD-L1^+^ variant displayed significantly lower miR-17-5p levels than the PD-L1^-^ counterpart (Figure 6A). On the contrary, miR-155-5p and miR-425-5p did not shown any apparent modulation in BiR or MiR cell lines (Supplementary Figure 2A-2B). Pearson's correlation between *CD274*/PD-L1 mRNA and miR-17-5p levels was r=-0.82, with the A375/BiR and /MiR cells clustering together. Similar results were obtained with the SKMEL5 cells, with r=-0.62. M14 cells behaved differently, with /BiR cells clustering with the sensitive cells, while M14/MiR appeared to express high levels of *CD274*/PD-L1 mRNA and low levels of miR-17-5p, with r=-0.87. This correlation was confirmed when studying miR-17-5p levels in A375/PD-L1^+^ and A375/PD-L1^-^ constitutive variants, with r=-0.80 (Figure 6B).

Transient transfection of pre-miR-17-5p in A375 and SKMEL5 /BiR and /MiR cell lines was followed by a sharp increase in intracellular miR17-5p levels, while at the same time *CD274*/PD-L1 mRNA levels decreased (Supplementary Figure 3A and Figure 6C). As expected, the drop in mRNA levels was accompanied by decreased in protein expression, as determined by western blot (Figure 6D) and by flow cytometry (Supplementary Figure 3B).

To determine whether miR-17-5p directly binds to the 3’-UTR region of PD-L1 we cloned part of 3’-UTR of PDL1 and expressed it in a luciferase reporter vector. We then co-transfected the reporter vector and miR-17-5p in 293T cells and observed a decrease in the luciferase activity after 48 hours, suggesting a direct effect (Figure 6E). Mutagenesis of miR-17-5p binding site in the 3’-UTR of PD-L1 mRNA (Figure 6F) failed to modulate luciferase activity, confirming specificity of binding between miR-17-5p and the 3’-UTR of PD-L1 mRNA (Figure 6E).

**Figure 7:**
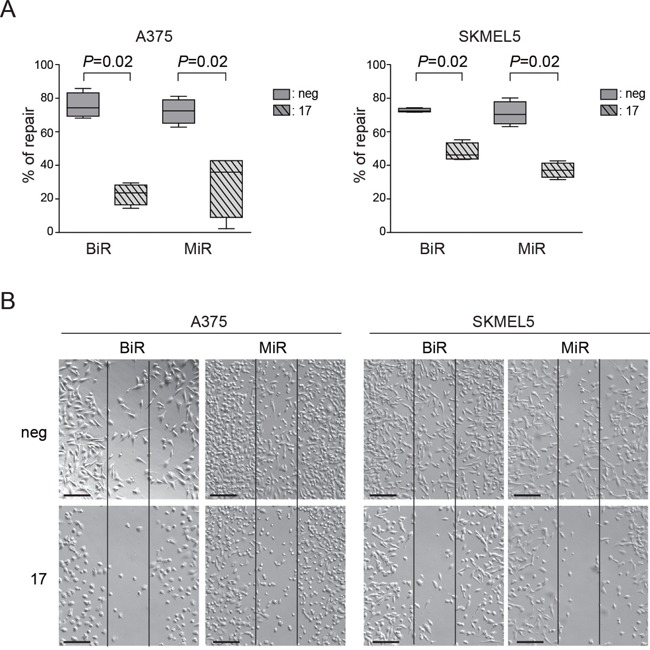
Modulation of PD-L1 can alter the functional properties of tumor cells **A**. Quantification as percentage of repair of wound-healing assay comparing miR-negative transfected control and miR-17-5p transfected A375/BiR, A375/MiR, SKMEL5/BiR and SKMEL5/MiR. **B**. Representative pictures (x10 magnification, scale bar 200 μm) of A375/BiR, A375/MiR, SKMEL5/BiR and SKMEL5/MiR.

In agreement with results obtained after silencing PD-L1, transfection of miR-17-5p in BiR and MiR melanoma cells was followed by a markedly decreased ability to repair wounds, indirectly validating the role of PD-L1 in this process (Figure 7A-7B). In these experimental conditions, miR17-5p levels rose sharply upon transfection (Supplementary Figure 3A).

### Expression of PD-L1 is acquired by BRAFi-resistant melanoma lesions, is associated to aggressive behavior and unfavorable outcome and is inversely correlated with plasmatic miR-17-5p levels

To obtain an independent validation of our *in vitro* data, we took advantage of a cohort of 80 BRAF^V600E^-mutated MM patients, treated with BRAFi. Patient characteristics are reported in Supplementary Table 3. For each of these patients PD-L1 expression, as well as tumor infiltration by mononuclear cells (TIMC), were determined by immunohistochemistry. Data are available for 78 patients, because one PD-L1^+^ patient was not evaluable for TIMC and one patient without TIMC was not evaluable for PD-L1 expression.

Four different patient subsets were defined based on the combination of the two parameters. Of the 28/78 patients with a PD-L1^+^ biopsy, 15 showed evidence of TIMC, suggesting that environmental factors, such as IFNγ produced by activated T cells, could be responsible for PD-L1 expression by the tumor cells. On the contrary, 13/28 of PD-L1^+^ patients did not show TIMC, arguing in favor of a constitutive expression of PD-L1 in this subset. Of the 50/78 patients with a PD-L1^-^ biopsy 14/50 had TIMC, while 36/50 did not have TIMC.

Our previous analysis of this cohort indicated that expression of PD-L1 by tumor cells and TIMC were two independent predictors of response to therapy with BRAFi [27, 31]. We now updated results with a median follow-up of 27.4 months, by which time 45 (64%) patients had progressed and 46 (52.5%) had died. Overall, 55 (69%) patients had either progressed or died, while 49 (61.3%) patients reached a complete or partial response at one time during the course of their illness.

**Figure 8:**
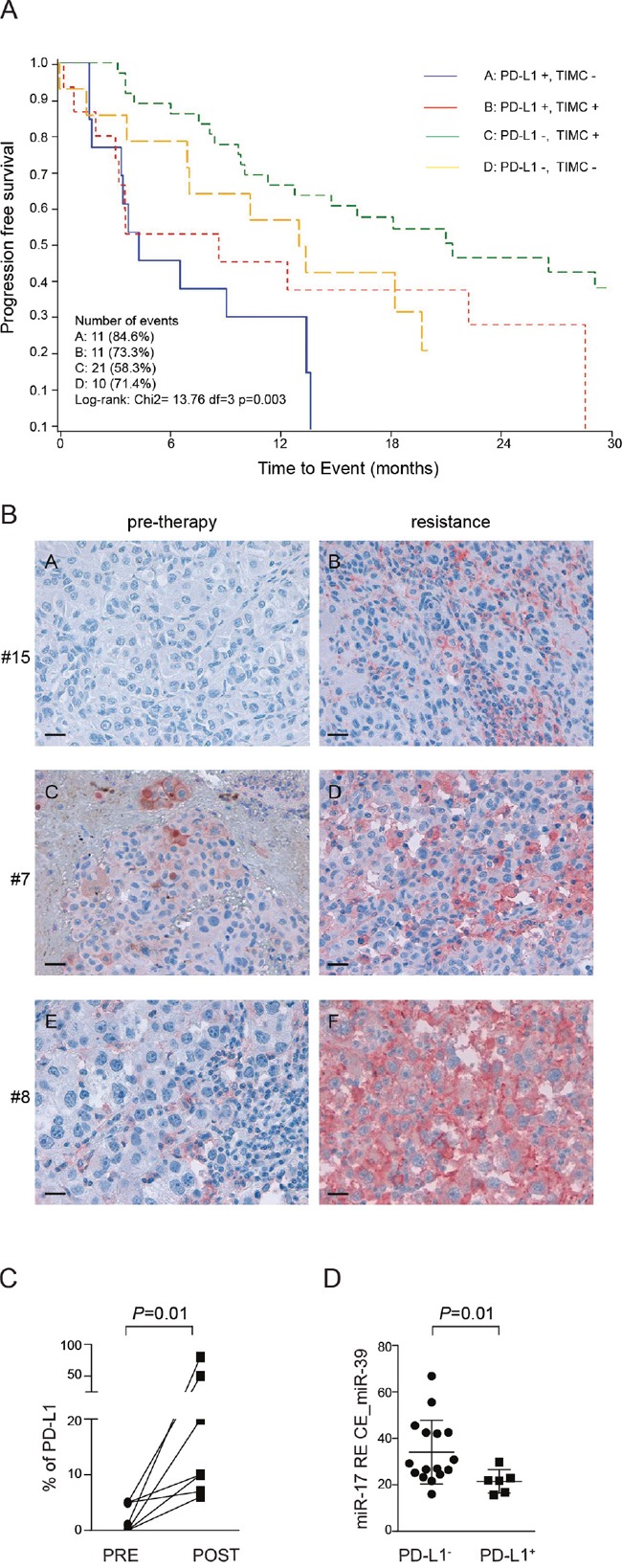
PD-L1 immunohistochemical expression in paired tissue biopsies taken before and after onset of resistance in patients treated with BRAF inhibitors (dabrafenib or vemurafenib) **A**. Survival curves estimated with the Kaplan-Meier method, on y axis is indicated the overall survival OV, on the x axis is indicated the time to event in months. **B**. PD-L1 expression in three representative patients (pre-therapy and upon resistance acquisition conditions): patient #15 (vemurafenib): PD-L1 is not expressed before resistance while it is observed in 6% of tumor cells after resistance; patient #7 (dabrafenib): PD-L1 positivity in 5% of tumor cells before resistance and in 10% of tumor cells upon resistance; patient #8 (dabrafenib): tumor tissue before resistance is considered PD-L1^-^, being observed only in 1% of tumor cells (cut-off ≥5%), while PD-L1 is strongly and diffusely expressed in 80% of tumor cells upon resistance. Original magnification x40, scale bar 50 μm. **C**. Percentage of PD-L1 immunostaining in tissue before resistance (PRE) and post resistance (POST) to BRAF inhibitors. **D**. Relative expression of miR-17-5p normalized on spike-in control miR-39 in serum blood patients before therapy.

Multivariate analysis indicated that patients with a PD-L1^+^ biopsy and without TIMC were the least likely to respond to treatment and consequently those with the shortest overall survival. This finding is in line with the hypothesis that constitutive PD-L1 expression by the tumor identifies a subset of MM patients characterized by a highly aggressive disease. Patients with PD-L1^−^ biopsies and without TIMC (OR 15.69, 95% CI 2.10–117.26, *P* < 0.0073) or patients with PD-L1^−^ biopsies, but with TIMC (OR 17.26, 95% CI 3.1–96.18, *P* < 0.0012) showed a higher probability to have a complete or partial response compared to patients with PD-L1^+^ biopsies and without TIMC, while patients with PD-L1^+^ biopsies and with TIMC showed an intermediate behavior (Figure 8A and Supplementary Table 4). Furthermore, after adjusting for stage and performance status, lack of PD-L1 expression and presence of TIMC were associated to a significantly longer progression free survival (PFS, HR 0.37, 95% CI 0.17–0.84, *P* < 0.02, Supplementary Table 5) and overall survival (OS, HR 0.35 95% CI 0.15–0.84, *P* < 0.02, Supplementary Table 6), compared to patients expressing PD-L1 and lacking TIMC. Kaplan Meier curves for PFS and OS are shown in Figure 8A and Supplementary Figure 4.

In order to evaluate whether PD-L1 could be dynamically modulated during BRAFi treatment, we obtained paired biopsies before beginning of therapy and after the onset of resistance to BRAFi (dabrafenib or vemurafenib) from 11 patients. Out of these 11 patients, in 7 cases (63%, 95% CI 39-94) we observed up-regulation of PD-L1 in melanoma cells by immunohistochemistry, as shown in three representative patients (Figure 8B-8C). Specifically, in 4 cases with PD-L1^-^ pre-treatment biopsies (failing to reach the ≥5% threshold) upon resistance we observed a shift from PD-L1^-^ to PD-L1^+^ status, while in the remaining cases PD-L1 was highly up-regulated in resistant melanoma tissues when compared with baseline, with diffuse and uniform membranous pattern of expression, irrespective from the presence of infiltrating immune cells (Figure 8B-8C and Supplementary Table 7).

Interestingly, we were able to obtain paired biopsies in 4 patients who previously received a BRAF inhibitor monotherapy (dabrafenib) alone, and subsequently a combination of dabrafenib and trametinib. In these patients, who progressed during BRAFi and MEKi, we did not observe any modulation of PD-L1 expression (not shown), in agreement with previous data [32].

Lastly, we asked whether miR-17-5p levels in plasma from patients with metastatic melanoma could be used as an inverse marker of the expression of PD-L1 by the tumor. We collected sera from patients with metastatic melanoma before treatment with BRAFi. Patients were divided into two groups according to PD-L1 expression obtained by IHC: following this approach 16 patients were PD-L1^+^ and 6 PD-L1^-^. RT-PCR was used to quantify miR-17-5p levels in patient sera, showing that patients with a PD-L1^+^ tumor had lower levels of miR17-5p (mean±SD 34.1±3.4), compared to patients with a PD-L1^-^ lesion (mean±SD 21.47±2.0, Figure 8D).

## DISCUSSION

The therapy of melanoma changed radically with the identification of recurrent mutations in genes coding for members of the MAPK pathway, which then led to the design of targeted inhibitors for both BRAF and MEK tyrosine kinases. Resistance to these drugs is a common event that is linked to the paradoxical activation of the MAP kinases axis, representing the limiting factor in this therapeutic approach. More recent studies are showing that the combination of tyrosine kinase inhibitors with immune checkpoint inhibitors, such as PD-L1, obtains more durable responses [33]. The rationale for these combinations is that MM frequently express PD-L1, which may be transcriptionally regulated by different signals. PD-L1 expression is dynamically modulated by IFN-γ, produced by infiltrating CD8^+^ activated T lymphocytes, representing an immune escape circuit. However, PD-L1 may be also expressed by melanoma cells in the absence of immune infiltrate, suggesting that expression relies on cell autonomous mechanisms.

The aim of this work was twofold. First, we wanted to expand our previous observations indicating that PD-L1 is a marker for an aggressive form of melanoma by addressing the issue of whether the molecule may be directly involved in determining this phenotype. Secondly, we wanted to understand how PD-L1 expression is regulated in these cells. The data obtained *in vitro* were then validated using a cohort of melanoma patients with BRAF mutations, treated with BRAF inhibitors and where PD-L1 expression in the metastatic lesions had been studied.

After stabilizing BiR and MiR cells, we observed that these variants had a more aggressive behavior compared to the S counterparts. Resistance induced expression of a panel of common genes linked to cell movement. Functionally, BiR and MiR cells were characterized by enhanced wound repair, which could be due to the up-regulation of molecules that play an essential role in melanoma metastasis, including integrin family members. However, a direct role of PD-L1 was shown after observing decreased wound healing in BiR or MiR cells where PD-L1 had been stably silenced by lentiviral infection. Interestingly, these cells also showed a marked down-regulation in the expression of CD56, which was proposed as a marker of desmoplastic and spindle-cell melanomas. Even if rare, this is a kind of melanoma with highly invasive properties. Even if future studies are needed to determine whether these tumors are constitutively PD-L1^+^, a recent report suggests that they are particularly sensitive to therapy with anti-PD1/PD-L1 antibodies (abstract ASCO 2015).

The second result of this work concerns the identification of a post-transcriptional regulation of PD-L1 levels, obtained through the miR-17-5p circuit. Our data indicate direct binding of this microRNA to the 3’-UTR of PD-L1. Furthermore, transfection of miR-17-5p decreases expression of PD-L1, as well as aggressiveness of the tumor. The role of miR-17-5p cluster in cancer is matter of intense investigation. Loss of heterozigosity at the miR-17-5p locus, has been observed in association with progression of different kinds of solid tumors [34]. Furthermore, the miR-17-92 cluster is deleted in a significant proportion of human cancers, including 20% of melanomas [35], suggesting an oncosuppressor role for this molecule. Our results would agree with this hypothesis, as BiR or MiR MM cell lines show a down-regulation of this microRNA, as compared to sensitive cells. The molecular circuits responsible for miR-17-5p modulation remain to be determined. At the light of recent data indicating loss of PTEN in aggressive melanoma, characterized by impaired immune responses, it is tempting to speculate that loss of PTEN may also be followed by loss of miR-17-5p, with the consequent lack of post-transcriptional regulation in PD-L1 and the described over-activation of the Wnt-β catenin Akt/PI3K pathways.

In the final part of the work, we exploited a large Italian cohort of MM patients that were treated with BRAFi or MEKi. From this analysis we draw three conclusions. The first is a confirmation that PD-L1 is an independent negative prognostic marker. The second is that, among PD-L1^+^ patients those lacking TIMC are characterized by the worst outcome, suggesting that constitutive PD-L1 expression characterizes the most aggressive form of the disease. The third is that the acquisition of resistance to BRAFi induces expression of PD-L1 in the majority of cases. Lastly, comparison of miR-17-5p levels in plasma of patients with PD-L1^+^ MM biopsies show decreased plasmatic levels of miR-17-5p compared to patients with a PD-L1^-^ lesion. If confirmed in larger cohorts, this finding suggests that miR-17-5p plasmatic levels may be used as inverse indicators of PD-L1 expression levels by the tumor. It will also be important to determine miR-17-5p levels in sequential samples and to correlate them with PD-L1 expression levels in the corresponding biopsies.

## MATERIALS AND METHODS

### Melanoma cell lines

The A375, SKMEL5 and M14 BRAF^V600E^-mutated cell lines were originally obtained through the American Type Culture Collection (ATCC) and the BRAF mutational status confirmed by Sanger sequencing. Cells were cultured in RPMI-1640 + 10% fetal calf serum and 100 IU/ml penicillin / streptomycin (all from Sigma, Milan, Italy, referred to as complete medium). BRAFi- and MEKi-resistant melanoma cells (indicated as BiR and MiR) were generated by treating cells with increasing concentrations of BRAFi (dabrafenib, GlaxoSmithKline, Brentford, UK) or MEKi (pimasertib, Merk Serono, Darmstadt, Germany), reaching the final concentration of 1.6 μM in ≈12 weeks. Cells were thereafter maintained under these culture conditions [23, 36].

### MTT viability assay

Cells were plated in complete medium and left overnight to attach to 96-well plates. BRAFi or MEKi were then added at the indicated concentrations for 72 hours, before a 4-hour incubation with 10 μl of (3-(4,5-dimethylthiazolyl-2)-2,5 diphenyltetrazolium bromide, MTT, Thermo Scientific, Milan, Italy). The formazan crystals formed were dissolved in sodium dodecyl sulfate-hydrochloride and absorbance was read at 570 nm using a microplate reader (Bio-Rad, Milan, Italy).

### Flow-cytometry

Antibodies used for flow cytometry were: anti-PD-L1-PE and -PE-Cy7, anti-CD24-APC, anti-CD56-PE, anti-HLA-DR-PE-Cy7 (all from eBioscience, Milan, Italy), anti-integrin-α3 mAb (from Prof. G. Tarone, University of Turin, Italy). A secondary PE-conjugated goat anti-mouse Ig (Thermo Scientific, Milan, Italy) was used to highlight the binding of unlabeled antibodies.

Immunofluorescence data were acquired using a FACSCantoII cytofluorimeter and processed with DIVA v8.0 (BD Biosciences) and FlowJo Version 9.01 softwares (TreeStar, Ashland, OR, USA), analyzing at least 10,000 events per sample.

### Western blot analysis

Cells were lysed, resolved by SDS-PAGE, and transferred to nitrocellulose filter membranes (Biorad, Milan) [27]. After blocking, membranes were incubated with: anti-PD-L1 (R&D Systems, Milan, Italy), -pSTAT3, -STAT3, -pERK1/2 and -ERK1/2 (all from Cell Signaling Technology, Danvers, MA). After incubation with horseradish peroxidase-conjugated secondary antibody (PerkinElmer, Milan, Italy), reaction was visualized with ECL using ImageQuant LAS4000 (GE Healthcare, Milan, Italy).

### Confocal microscopy

Cells were cultured overnight on glass cover slips in 24-well plates before incubation with unlabeled anti-PD-L1 antibody (eBioscience), followed by AlexaFluor-488-conjugated goat anti-mouse IgG (Thermo Scientific). Phalloidin AlexaFluor-568-conjugated and DAPI (both from Thermo Scientific) were added after fixation (4% paraformaldehyde) and permeabilization (0.1% saponin). Slides were then analyzed using a TCS SP5 laser scanning confocal microscope equipped with 4 lasers and images were acquired with LAS AF Version Lite 2.4 software (Leica Microsystems, Wetzlar, Germany), as described [37]. Files were processed with Photoshop (Adobe Systems, San Jose, CA).

### Wound healing assay

Melanoma cells were seeded in 6-well plates (5 × 10^5^/well) and incubated in complete medium overnight. Cells were then treated with Mitomycin C (10 μg, 20 minutes, Sigma). Wounds were made with a 200 μl tip and the wells were washed several times to remove all non-adherent cells. Wound repair was documented at 24 hours using a DMI 3000 B optical microscope (Leica Microsystems), equipped with a DCF 310 FX digital camera and LAS Version 3.8 software. Images were analyzed with MRI Wound Healing Tool of ImageJ software (NIH, USA). The percentage of repair was calculated as: [(area _24 hours_/area_0 hours_) × 100%].

### Chemotaxis and invasion assays

Migration and invasion were measured using 8 μM pore Boyden chambers (Corning, Corning, NY). Briefly, 10^5^ cells were plated in the upper chamber in serum free RPMI-1640 medium, while complete medium was added as a chemoattractant in the lower part. After 4 hours, cells in the upper part of the chamber were removed, while cells that had migrated to the lower surface of the filter stained with crystal violet (Sigma) and analyzed by bright-field microscopy. Migration index was calculated as: number of cells migrated in the presence of the chemoattractant / number of cells migrated without chemoattractant.

Invasion assays were performed after covering the upper part of the well with Matrigel (0.5 mg/ml, Corning). After 24 hours, cells that had not penetrated were wiped away, while cells that had invaded the lower surface of the filter were stained with crystal violet and examined by bright-field microscopy [38]. Invasion index was calculated as: number of cells penetrated in the presence of the chemoattractant / number of cells penetrated without chemoattractant.

### Xenograft models

A375/S and A375/BiR cells (10^7^) were injected subcutaneously in the presence of Matrigel into the right and left flanks, respectively, of 6- to 8-week-old male NOD/SCID mice. When tumors became palpable (≈ after two weeks), mice were treated with BRAFi (30 mg/kg) daily by gavage for 1 week (Tafinlar, Novartis, Basel, Switzerland). After treatment, animals were sacrificed and tumors measured. Lesions were then partly fixed and processed for histopathological studies and partly dissociated for cytofluorimetric analyses and cultures. Re-cultured cells were tested to confirm resistance to BRAFi by the MTT assay.

### Quantitative real-time PCR (qRT-PCR)

Total RNA (containing mRNAs and microRNAs) was extracted using miRNeasy kit (Qiagen, Milan, Italy) and converted to cDNA using the High Capacity cDNA Reverse Transcription kit (Thermo Scientific). qRT-PCR was performed using the 7900 HT Fast Real Time PCR system (SDS2.3 software) using commercially available primers (all from Thermo Scientific) and standardized over actin levels. Reactions were done in triplicate from the same cDNA reaction (technical replicates). Detection of microRNAs was performed by qRT-PCR for the specific hsa-miR and U6 snRNA (ID 001973, Thermo Scientific), as control for the cell lines. For normalization in blood sera from patients miR-39 from *C. elegans* [39] was added during the extraction of microRNAs. The comparative CT method was used to calculate the relative expression of the gene under analysis.

TargetScan 6.0 algorithm was used to identify predicted microRNA targets that bind 3’-UTR of PD-L1 mRNA.

### RNA sequencing (RNA-seq)

RNA-seq was performed as previously described [40], with few modifications. Briefly, the DNF-471 Standard Sensitivity RNA Analysis Kit, run on Fragment Analyzer (both from Advanced Analytical, Ankeny IA) was used to check RNA quality. Libraries were prepared from total RNA using TruSeq RNA Sample Preparation v2 according to the manufacturer's protocol (Illumina, San Diego, CA). Samples were sequenced on Illumina HiScanSQ platform. Sequencing reads were trimmed out of the low-quality bases with Fastx Toolkit (HannonLab, CHSL). Filtered sequences were mapped on hg19 genome assembly by using TopHat v2.0.6 and mRNA quantification was performed using Cuffdiff v2.0.2. For downstream analysis, genes with RPKM < 1 in all the samples were filtered out. Custom scripts on R software were used for clustering and heatmap analysis (https://www.r-project.org/). Gene Ontology was analyzed by using Database for Annotation, Visualization and Integrated Discovery (DAVID) program (https://david.ncifcrf.gov/).

### Transient transfections

Melanoma cell lines were transfected with pre-miR-17-5p microRNA precursor molecules and microRNA negative control (Thermo Scientific) using HiPerFect Transfection Reagent (Qiagen). Expression of miRs or protein-coding genes overexpression/knockdown was monitored from 24 to 96 hours later by qRT-PCR.

### Luciferase assays

Luciferase reporter vectors containing the partial PD-L1 3’-UTR were generated following PCR amplification (forward primer: CAGGCAAGAATTGTGGCTGA, reverse primer: CCAAGTAACTTTCTCCACTGGGAT) of the 3’-UTR from human genomic DNA of PD-L1^+^ cells and cloning into the Firefly Luciferase reporter pMIR REPORT™ luciferase vector (Thermo Scientific). When indicated the 3’-UTR was mutagenized at the miR-17-5p recognition site using the QuickChange Site-Directed Mutagenesis kit (Stratagene, Cedar Creek, TX) according to the manufacturer's instructions (forward primer: GATGAAACATGAGACAAAAGGGATTATGAGGCTT ACAAATCCAACACCACAAGGA, reverse primer: TCCTTGTGGTGTTGGATTTGTAAGCCTCATAATCCC TTTTGTCGCATGTTTCATC).

Cells (5 × 10^4^) were then co-transfected with 50 ng of the pMIR REPORT™ (Thermo Scientific) Firefly Luciferase constructs containing the 3’-UTRs of the specific microRNA potential target, 20 ng of pRL-TK Renilla Luciferase normalization control (Promega, Madison, WI) and 75 nM of the indicated pre-miR using Effectene (Qiagen). Lysates were collected 48 hours after transfection and Firefly and Renilla Luciferase activities were measured with a Dual-Luciferase Reporter System (Promega).

### Preparation of lentiviruses

Lentiviral particles containing the genetic material for shPD-L1 (Origene, Rockville, MD) were generated according to the manufacture's protocol.

Resistant cell lines were infected with shPD-L1 lentiviral particles and expression of the molecule monitored 48 hours later by flow cytometry. To obtain stably PD-L1-silenced clones from A375 or SKMEL5 /BiR or /MiR lines, infected cells were repeatedly sorted with a BD FACSAriaIII (BD Biosciences) by gating on GFP^+^/PD-L1^-^.

### PD-L1 immunohistochemistry in human melanoma tissues

Cohort characteristics and detailed immunohistochemical protocols were previously described [31].

### Statistical analyses

Continuous variables were compared by Mann-Whitney test. The Wilcoxon matched-pairs signed rank test was used for paired variables.

For the clinical study, all melanoma patients satisfying eligibility criteria and treated with BRAFi were considered for analysis. Overall response rate (ORR) was defined as the proportion of patients with complete response or partial response, according to RECIST, v1.1 [41]. Progression free survival (PFS) was defined as the time from the beginning of BRAFi to first appearance of progressive disease or death for any cause; patients known to be alive and without progressive disease at the time of analysis were censored at their last available follow-up assessment. Overall survival (OS) was defined as the time from the beginning of BRAFi to the date of death from any cause or the date of the last follow-up. Survival curves were estimated with the Kaplan-Meier method. PFS and OS were analyzed by means of Cox regression model and results were expressed as hazard ratios (HR) with their 95% confidence intervals (95% CI) ORR was analyzed by means of logistic regression models and results were expressed as odds ratios (OR) with their 95 %CI.

Statistical analyses were performed using SAS software, version 9.2 (SAS Institute, Cary, NC) and GraphPad version 6 (GraphPad Software Inc, La Jolla, CA).

## SUPPLEMENTARY FIGURES AND TABLES




