# Supplementary material for: PD-L1 up-regulation in melanoma increases disease aggressiveness and is mediated through miR-17-5p
Source: Oncotarget. 2017 Feb 9;8(9):15894–911. doi: 10.18632/oncotarget.15213 (PMC5362532; doi:10.18632/oncotarget.15213)
Supplement: Supplementary file 1 [file oncotarget-08-15894-s001.pdf]

PD-L1 up-regulation in melanoma increases disease aggressiveness and is mediated through miR-17-5p

SUPPLEMENTARY FIGURES AND TABLES

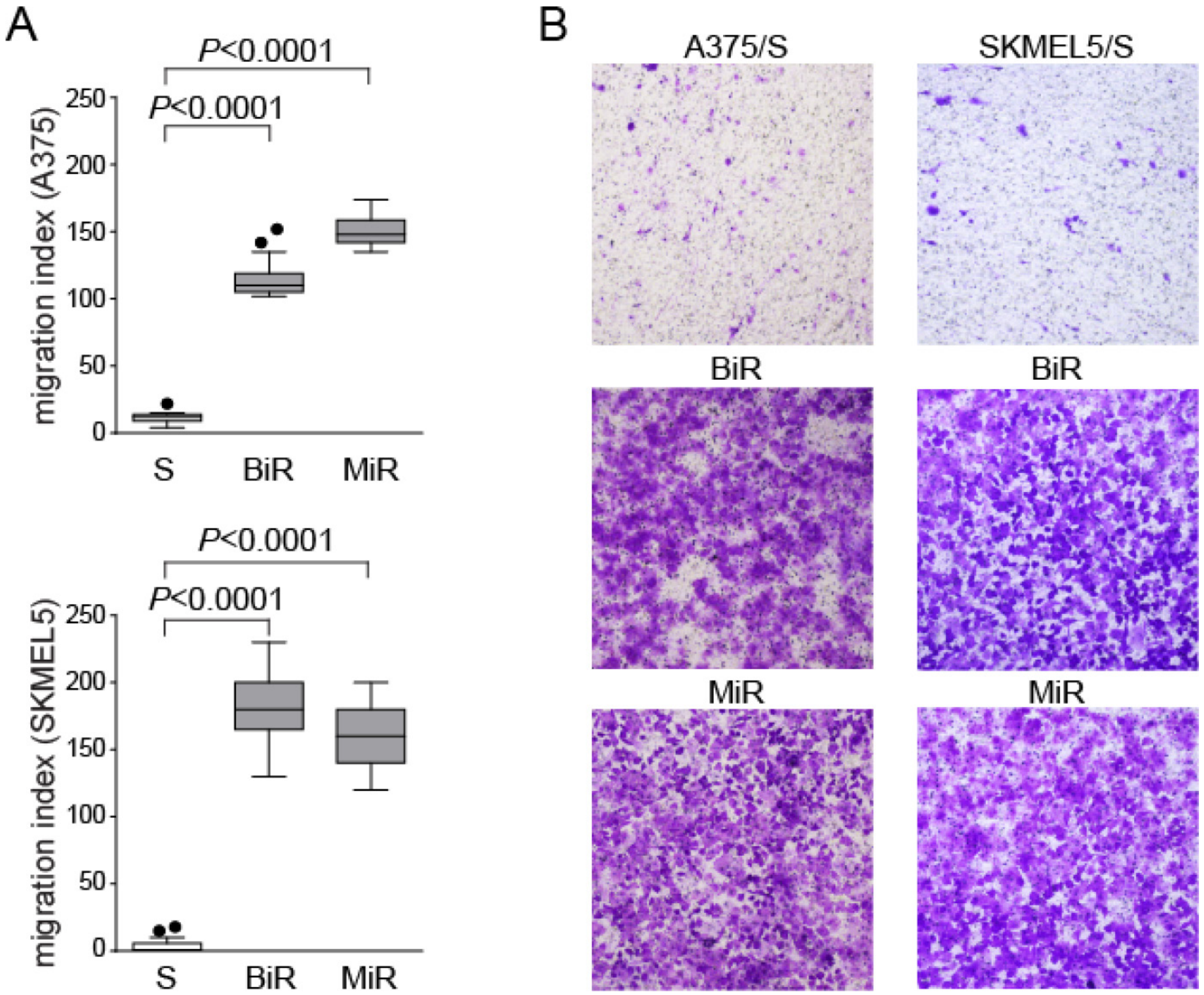

Supplementary Figure 1: Over-expression of PD-L1 in BRAFi- and MEKi-resistant cell lines contributes to increased chemotaxis. **A.** Box plots representing cumulative data of migration assays in A375 and SKMEL5 cell variants. **B.** Representative images (x10 magnification) of A375 (left panels) and SKMEL5 (right panels) cell lines comparing S, BiR and MiR variants.

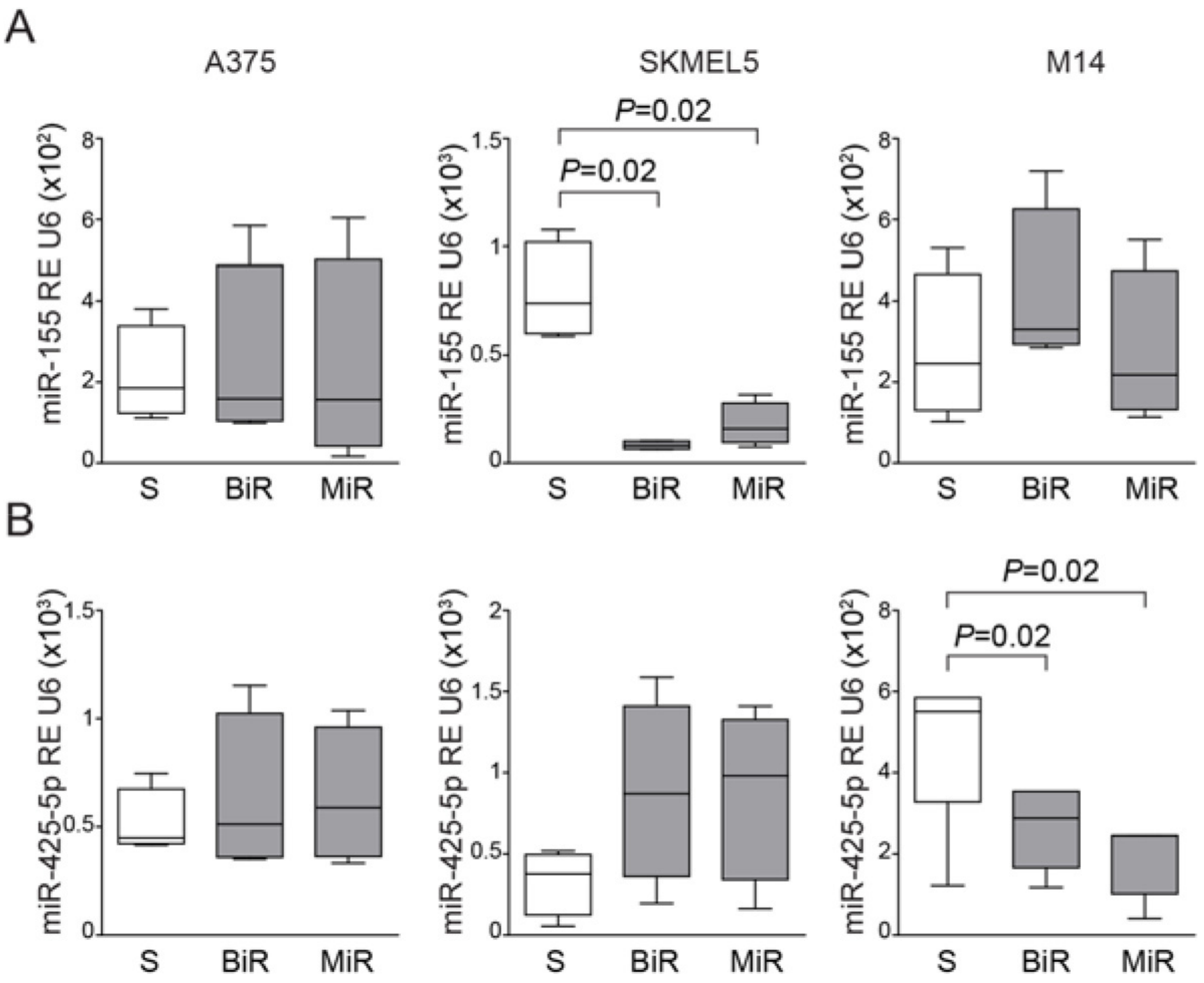

Supplementary Figure 2: Identification of PD-L1 post-transcriptional regulators. A. miR-155 and **B.** miR-425-5p expression level relative to U6 of sensitive and resistant A375, SKMEL5 and M14 cell lines.

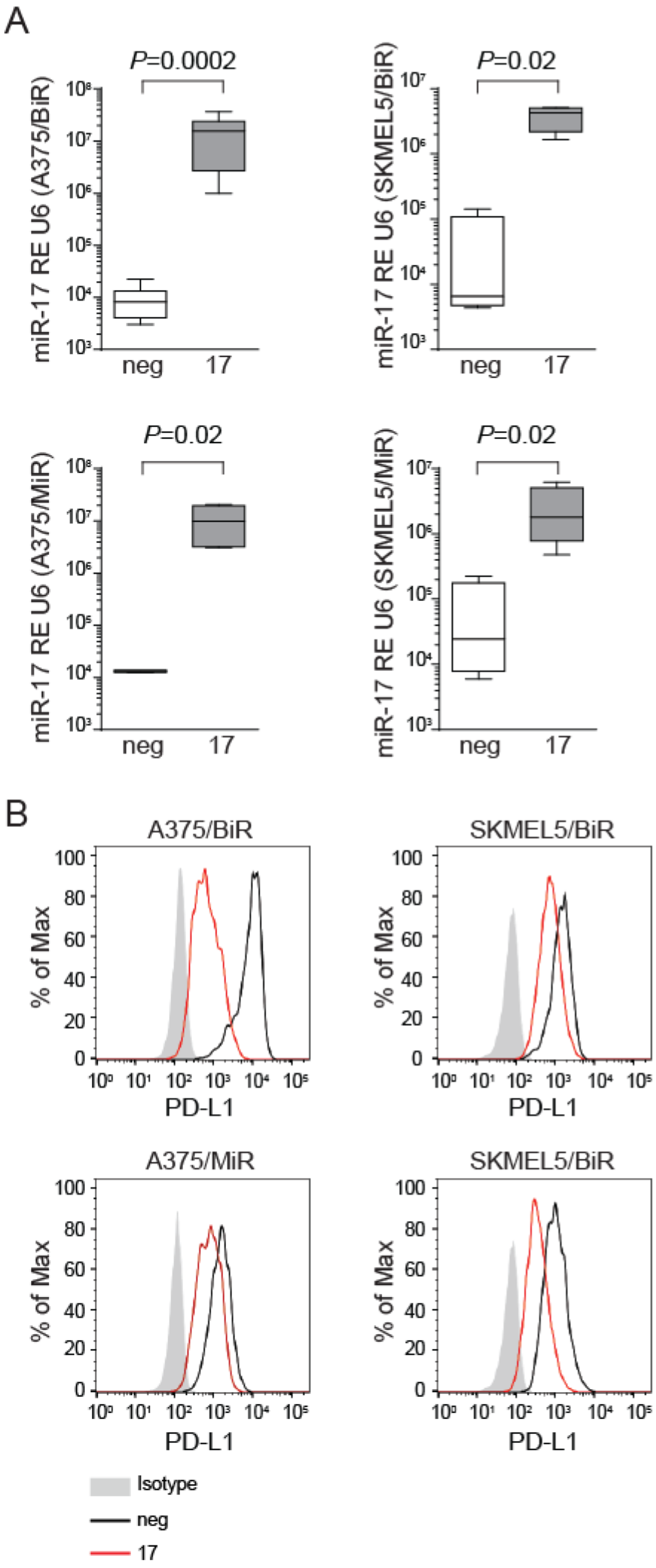

Supplementary Figure 3: Transient transfection of pre-miR17-5p in BRAFi- and MEKi-resistant cell lines regulates PD-L1 expression. A. RT-PCR analysis of miR-17 expression relative to U6 in A375 and SKMEL5 variants. **B.** Histogram representing PD-L1 expression comparing BRAFi- and MEKi-resistant A375 and SKMEL5 cells following transfection with miR-negative control (neg) and pre-miR17-5p (17).

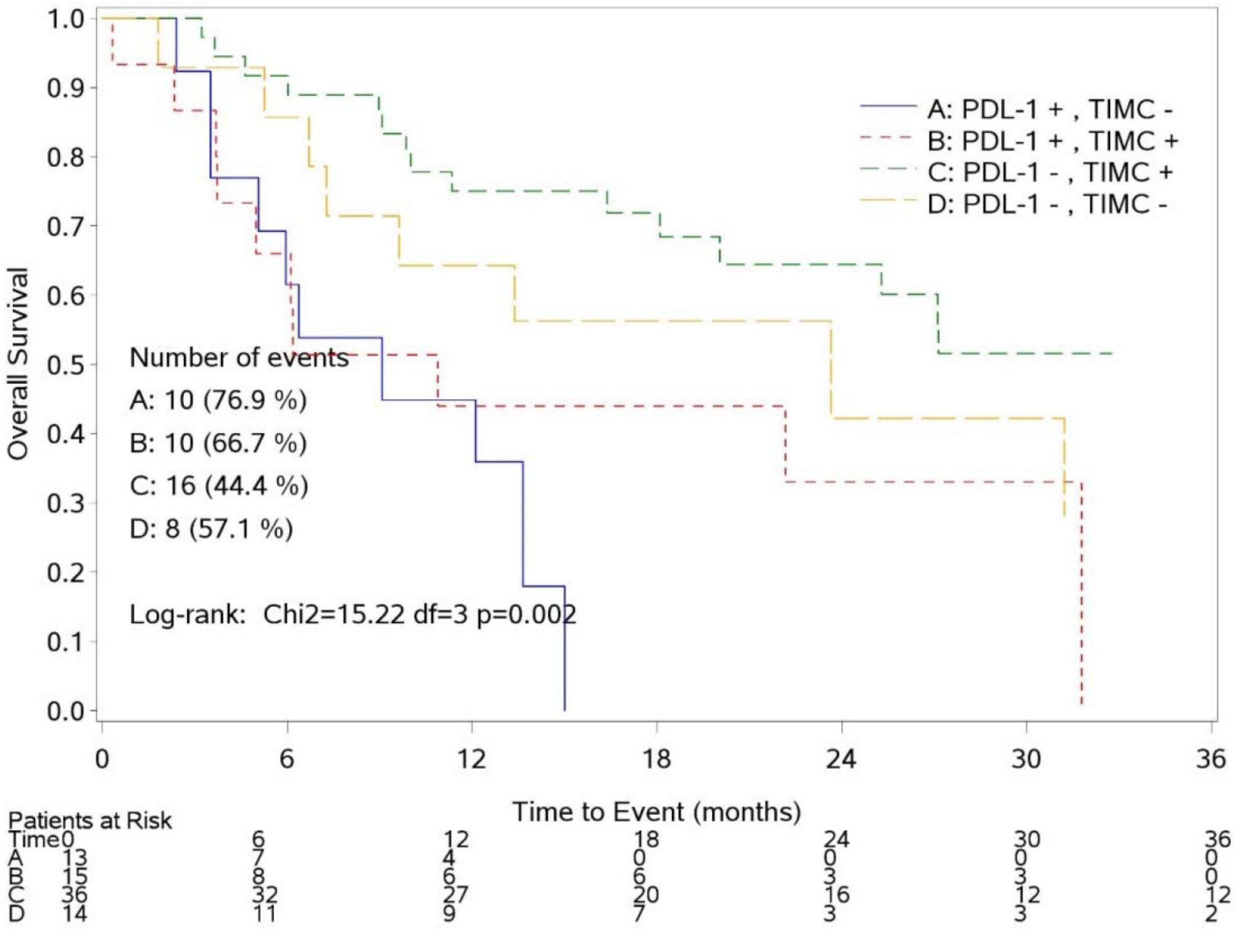

Supplementary Figure 4: Overall Survival – KM curves for PDL-1/TIMC.

Supplementary Table 1: 206 differentially expressed common genes comparing A375 (BiR vs S) and A375 (PD-L1^{+} vs PD-L1^{-})

See Supplementary File 1

Supplementary Table 2: 852 differentially expressed common genes (816 annotated listed in the table) comparing A375 (BiR/MiR vs S) and SKMEL5 (BiR/MiR vs S). These genes define the resistance signature

See Supplementary File 1

Supplementary Table 3: Patients characteristics

	n (%)
**Sex**	
Female	38 (47.5 %)
Male	42 (52.5%)
**Age at diagnosis**	
Median (q1-q3)	56.2 (44.6-68.0)
**Age at start therapy (BRAFi)**	
Median (q1-q3)	58.2 (48.6-70.2)
**Stage**	
M1a	33 (41.2%)
M1b	3 (3.8%)
M1c	44 (55.0%)
**Performance Status**	
0	52 (65.0%)
1	28 (35.0%)
**Previous therapies**	
Yes	8 (10.0%)
No	72 (90.0%)
**Number of previous therapies (n=8)**	
1*	5 (62.5%)
>1**	3 (37.5%)
**Therapies post BRAFi**	
Yes	22 (27.5%)
Immunotherapy	13 (59.1%)
other	9 (40.9%)
No	58 (72.5%)
**Number of therapies post BRAFi (n=22)**	
1***	19 (86.4%)
>1****	3 (13.6%)

Legend.
* therapies received: Fotemustina; Dacarbazina; Ipilimumab; Temozolamide; CVD.
** therapies received: Dacarbazina, Ipilimumab; CDDP+Deticene, Ipilimumab; Dacarbazina, Fotemustina, Paclitaxel.
*** therapies received: 10 patients: Ipilimumab; 5 patients: Dacarbazina; 2 patients: Fotemustina; 1 patient: CVD; 1 patient: Ipilimumab + antiPD-1.
**** therapies received: Ipilimumab, Nivolumab; Ipilimumab, carboplatino + taxolo, dacarbazina; dacarbazina, fotemustina.

Supplementary Table 4: Logistic model - Multivariate analysis – event =SD/PD

	OR	95% CI		p-value
Stage (M1c vs M1b vs M1a)	0.66	0.37	1.19	0.1707
PDL-1 + , TIMC -		reference		
PDL-1 + , TIMC +	3.79	0.60	24.09	0.1575
PDL-1 - , TIMC +	17.26	3.10	96.18	**0.0012**
PDL-1 - , TIMC -	15.69	2.10	117.26	**0.0073**

Legend.
PDL1. Programmed death-ligand 1, TIMC: **tumor-infiltrating immune cells.**

Supplementary Table 5: Cox models – PFS - multivariate models

	HR	95% CI		p-value
Stage (M1c vs M1b vs M1a)	1.66	1.19	2.31	**0.0027**
PS (1 vs 0)	1.80	0.96	3.37	0.0657
PDL-1 + , TIMC -		reference		
PDL-1 + , TIMC +	0.80	0.33	1.97	0.6285
PDL-1 - , TIMC +	0.37	0.17	0.84	**0.0176**
PDL-1 - , TIMC -	0.73	0.30	1.79	0.4875

Legend.
PDL1. Programmed death-ligand 1, TIMC: **tumor-infiltrating immune cells.**

Supplementary Table 6: Cox models – OS - multivariate models

	HR	95% CI		p-value
Stage (M1c vs M1b vs M1a)	1.70	1.17	2.48	**0.0054**
PS (1 vs 0)	2.70	1.38	5.30	**0.0038**
PDL-1 + , TIMC -		reference		
PDL-1 + , TIMC +	0.96	0.38	2.42	0.9222
PDL-1 - , TIMC +	0.35	0.15	0.84	**0.0182**
PDL-1 - , TIMC -	0.61	0.22	1.64	0.3245

Legend.
PDL1. Programmed death-ligand 1, TIMC: **tumor-infiltrating immune cells, PS: Performance status.**

Supplementary Table 7: IHC staining for PD-L1 expression in paired biopsies of MM patients

Patient ID #	Date of birth	Therapy	PD-L1 immunostaining in tissue before resistance (%)	PD-L1 immunostaining in tissue after resistance (%)
1	06/10/57	Vemurafenib	0	0
6	10/01/48	Vemurafenib	4	4
7	17/05/05	Dabrafenib	5	10
8	19/07/64	Dabrafenib	1	80
9	27/06/42	Vemurafenib	0	0
11*	31/12/45	Dabrafenib and Trametinib	0	0
15	15/09/76	Vemurafenib	0	6
18*	30/01/65	Vemurafenib and Cobimetinib	0	0
21	13/06/63	Dabrafenib	5	7
23*	02/01/72	Dabrafenib and Trametinib	0	0
26	13/01/64	Vemurafenib	0	20
28	30/05/62	Vemurafenib	5	5
39	17/09/52	Vemurafenib	5	50
40	27/12/33	Vemurafenib	0	10
46*	01/03/64	Dabrafenib and Trametinib	0	0

PD-L1 up-regulation in paired biopsies obtained before and after onset of resistance in 11 patients receiving a BRAF inhibitors (Dabrafenib or Vemurafenib) and in 4* patients treated with BRAF and MEK inhibitors.
* Patients treated with BRAF (dabrafenib) and MEK (trametinib) inhibitors.
Legend: IHC: Immunohistochemical.
