# Supplementary material for: PD-L1 up-regulation in melanoma increases disease aggressiveness and is mediated through miR-17-5p
Source: Oncotarget. 2017 Feb 9;8(9):15894–911. doi: 10.18632/oncotarget.15213 (PMC5362532; doi:10.18632/oncotarget.15213)
Supplement: Supplementary file 2 [file oncotarget-08-15894-s002.docx]

Supplemental data

**Supplementary Table 1. 206 differentially expressed common genes comparing A375 (BiR vs S) and A375 (PD-L1^+^ vs PD-L1^-^).**

|  |
| --- |
| **OFFICIAL_GENE_SYMBOL Name Species** |
| ABCA1 ATP-binding cassette, sub-family A (ABC1), member 1 Homo sapiens |
| ABCC3 ATP-binding cassette, sub-family C (CFTR/MRP), member 3 Homo sapiens |
| ACO1 aconitase 1, soluble Homo sapiens |
| ACPL2 acid phosphatase-like 2 Homo sapiens |
| ACTA2 actin, alpha 2, smooth muscle, aorta Homo sapiens |
| ADA adenosine deaminase Homo sapiens |
| ALCAM hypothetical protein LOC100133690; activated leukocyte cell adhesion molecule Homo sapiens |
| AMOT angiomotin Homo sapiens |
| ARHGAP12 Rho GTPase activating protein 12 Homo sapiens |
| ARNT2 aryl-hydrocarbon receptor nuclear translocator 2 Homo sapiens |
| ASAP3 ArfGAP with SH3 domain, ankyrin repeat and PH domain 3 Homo sapiens |
| ASS1 argininosuccinate synthetase 1 Homo sapiens |
| ATF3 activating transcription factor 3 Homo sapiens |
| B4GALNT1 beta-1,4-N-acetyl-galactosaminyl transferase 1 Homo sapiens |
| BASP1 brain abundant, membrane attached signal protein 1 Homo sapiens |
| BCL6 B-cell CLL/lymphoma 6 Homo sapiens |
| BLVRB biliverdin reductase B (flavin reductase (NADPH)) Homo sapiens |
| BMP2 bone morphogenetic protein 2 Homo sapiens |
| BTBD11 BTB (POZ) domain containing 11 Homo sapiens |
| C10orf47 chromosome 10 open reading frame 47 Homo sapiens |
| CADM1 cell adhesion molecule 1 Homo sapiens |
| CAPS calcyphosine Homo sapiens |
| CAV2 caveolin 2 Homo sapiens |
| CCL5 chemokine (C-C motif) ligand 5 Homo sapiens |
| CD109 CD109 molecule Homo sapiens |
| CD274 CD274 molecule Homo sapiens |
| CD68 CD68 molecule Homo sapiens |
| CD74 CD74 molecule, major histocompatibility complex, class II invariant chain Homo sapiens |
| CDK2AP1 cyclin-dependent kinase 2 associated protein 1 Homo sapiens |
| CHST7 carbohydrate (N-acetylglucosamine 6-O) sulfotransferase 7 Homo sapiens |
| CHSY1 chondroitin sulfate synthase 1 Homo sapiens |
| CITED2 Cbp/p300-interacting transactivator, with Glu/Asp-rich carboxy-terminal domain, 2 Homo sapiens |
| COL4A5 collagen, type IV, alpha 5 Homo sapiens |
| COL5A1 collagen, type V, alpha 1 Homo sapiens |
| CRELD2 cysteine-rich with EGF-like domains 2 Homo sapiens |
| CSPG4 chondroitin sulfate proteoglycan 4 Homo sapiens |
| CSRP2 cysteine and glycine-rich protein 2 Homo sapiens |
| CTDSPL CTD (carboxy-terminal domain, RNA polymerase II, polypeptide A) small phosphatase-like Homo sapiens |
| CTHRC1 collagen triple helix repeat containing 1 Homo sapiens |
| CTNNBIP1 catenin, beta interacting protein 1 Homo sapiens |
| CYP26B1 cytochrome P450, family 26, subfamily B, polypeptide 1 Homo sapiens |
| CYP27B1 cytochrome P450, family 27, subfamily B, polypeptide 1 Homo sapiens |
| CYR61 cysteine-rich, angiogenic inducer, 61 Homo sapiens |
| DNAJC12 DnaJ (Hsp40) homolog, subfamily C, member 12 Homo sapiens |
| DNER delta/notch-like EGF repeat containing Homo sapiens |
| DOCK2 dedicator of cytokinesis 2 Homo sapiens |
| DUSP5 dual specificity phosphatase 5 Homo sapiens |
| EHBP1 EH domain binding protein 1 Homo sapiens |
| ELF4 E74-like factor 4 (ets domain transcription factor) Homo sapiens |
| ELL2 elongation factor, RNA polymerase II, 2 Homo sapiens |
| ERBB3 v-erb-b2 erythroblastic leukemia viral oncogene homolog 3 (avian) Homo sapiens |
| ERO1LB ERO1-like beta (S. cerevisiae) Homo sapiens |
| FAM107B family with sequence similarity 107, member B Homo sapiens |
| FAM108C1 family with sequence similarity 108, member C1 Homo sapiens |
| FAM167A family with sequence similarity 167, member A Homo sapiens |
| FAM84B family with sequence similarity 84, member B Homo sapiens |
| FARP1 FERM, RhoGEF (ARHGEF) and pleckstrin domain protein 1 (chondrocyte-derived) Homo sapiens |
| FEZ1 fasciculation and elongation protein zeta 1 (zygin I) Homo sapiens |
| FGF2 fibroblast growth factor 2 (basic) Homo sapiens |
| FGFRL1 fibroblast growth factor receptor-like 1 Homo sapiens |
| FLJ20021 hypothetical LOC90024 Homo sapiens |
| FLNB filamin B, beta (actin binding protein 278) Homo sapiens |
| FLRT3 fibronectin leucine rich transmembrane protein 3 Homo sapiens |
| FOS v-fos FBJ murine osteosarcoma viral oncogene homolog Homo sapiens |
| FOSB FBJ murine osteosarcoma viral oncogene homolog B Homo sapiens |
| FST follistatin Homo sapiens |
| FSTL1 follistatin-like 1 Homo sapiens |
| FSTL3 follistatin-like 3 (secreted glycoprotein) Homo sapiens |
| FTH1 ferritin, heavy polypeptide 1; ferritin, heavy polypeptide-like 16 Homo sapiens |
| FZD8 frizzled homolog 8 (Drosophila) Homo sapiens |
| GAL3ST4 galactose-3-O-sulfotransferase 4 Homo sapiens |
| GAS6 similar to growth arrest-specific 6; growth arrest-specific 6 Homo sapiens |
| GDF15 growth differentiation factor 15 Homo sapiens |
| GFPT2 glutamine-fructose-6-phosphate transaminase 2 Homo sapiens |
| GLIPR2 GLI pathogenesis-related 2 Homo sapiens |
| GPNMB glycoprotein (transmembrane) nmb Homo sapiens |
| HBEGF heparin-binding EGF-like growth factor Homo sapiens |
| HEY1 hairy/enhancer-of-split related with YRPW motif 1 Homo sapiens |
| HHEX hematopoietically expressed homeobox Homo sapiens |
| HLA-DMA major histocompatibility complex, class II, DM alpha Homo sapiens |
| HLA-DPA1 major histocompatibility complex, class II, DP alpha 1 Homo sapiens |
| HLA-DRA major histocompatibility complex, class II, DR alpha Homo sapiens |
| HLA-DRB6 major histocompatibility complex, class II, DR beta 6 (pseudogene) Homo sapiens |
| HMGA2 high mobility group AT-hook 2 Homo sapiens |
| HMGB1 high-mobility group box 1; high-mobility group box 1-like 10 Homo sapiens |
| IFIT1 interferon-induced protein with tetratricopeptide repeats 1 Homo sapiens |
| IGF2BP3 insulin-like growth factor 2 mRNA binding protein 3 Homo sapiens |
| IGFBP4 insulin-like growth factor binding protein 4 Homo sapiens |
| IGFBP6 insulin-like growth factor binding protein 6 Homo sapiens |
| IGFBP7 insulin-like growth factor binding protein 7 Homo sapiens |
| IL11 interleukin 11 Homo sapiens |
| IL7R interleukin 7 receptor Homo sapiens |
| ING1 inhibitor of growth family, member 1 Homo sapiens |
| IRAK2 interleukin-1 receptor-associated kinase 2 Homo sapiens |
| IRS2 insulin receptor substrate 2 Homo sapiens |
| IRX3 iroquois homeobox 3 Homo sapiens |
| ITGA3 integrin, alpha 3 (antigen CD49C, alpha 3 subunit of VLA-3 receptor) Homo sapiens |
| ITGB2 integrin, beta 2 (complement component 3 receptor 3 and 4 subunit) Homo sapiens |
| KCNK1 potassium channel, subfamily K, member 1 Homo sapiens |
| KHDRBS3 KH domain containing, RNA binding, signal transduction associated 3 Homo sapiens |
| KIAA1715 KIAA1715 Homo sapiens |
| KRT81 keratin 81 Homo sapiens |
| LAMB3 laminin, beta 3 Homo sapiens |
| LDOC1 leucine zipper, down-regulated in cancer 1 Homo sapiens |
| LEF1 lymphoid enhancer-binding factor 1 Homo sapiens |
| LMO4 LIM domain only 4 Homo sapiens |
| LOC100127888 hypothetical protein LOC100127888 Homo sapiens |
| LOC645166 lymphocyte-specific protein 1 pseudogene Homo sapiens |
| LPAR1 lysophosphatidic acid receptor 1 Homo sapiens |
| LRIG1 leucine-rich repeats and immunoglobulin-like domains 1 Homo sapiens |
| LRRC20 leucine rich repeat containing 20 Homo sapiens |
| LY96 lymphocyte antigen 96 Homo sapiens |
| MAGEA1 melanoma antigen family A, 1 (directs expression of antigen MZ2-E) Homo sapiens |
| MAGEA10 melanoma antigen family A, 10 Homo sapiens |
| MAP1B microtubule-associated protein 1B Homo sapiens |
| MAP3K1 mitogen-activated protein kinase kinase kinase 1 Homo sapiens |
| MEOX2 mesenchyme homeobox 2 Homo sapiens |
| MFAP2 microfibrillar-associated protein 2 Homo sapiens |
| MGLL monoglyceride lipase Homo sapiens |
| MGP matrix Gla protein Homo sapiens |
| MMP1 matrix metallopeptidase 1 (interstitial collagenase) Homo sapiens |
| MSLN mesothelin Homo sapiens |
| MT2A metallothionein 2A Homo sapiens |
| MVP major vault protein Homo sapiens |
| MYH9 myosin, heavy chain 9, non-muscle Homo sapiens |
| NAB2 NGFI-A binding protein 2 (EGR1 binding protein 2) Homo sapiens |
| NCAM1 neural cell adhesion molecule 1 Homo sapiens |
| NDRG4 NDRG family member 4 Homo sapiens |
| NMNAT2 nicotinamide nucleotide adenylyltransferase 2 Homo sapiens |
| NR4A2 nuclear receptor subfamily 4, group A, member 2 Homo sapiens |
| NTN4 netrin 4 Homo sapiens |
| NTNG1 netrin G1 Homo sapiens |
| OAS2 2'-5'-oligoadenylate synthetase 2, 69/71kDa Homo sapiens |
| OBFC2A oligonucleotide/oligosaccharide-binding fold containing 2A Homo sapiens |
| OSCAR osteoclast associated, immunoglobulin-like receptor Homo sapiens |
| PALLD palladin, cytoskeletal associated protein Homo sapiens |
| PDE9A phosphodiesterase 9A Homo sapiens |
| PDIA4 protein disulfide isomerase family A, member 4 Homo sapiens |
| PINK1 PTEN induced putative kinase 1 Homo sapiens |
| PLP1 proteolipid protein 1 Homo sapiens |
| PMP22 peripheral myelin protein 22 Homo sapiens |
| PNMA2 paraneoplastic antigen MA2 Homo sapiens |
| POU3F2 POU class 3 homeobox 2 Homo sapiens |
| PRR4 proline rich 4 (lacrimal) Homo sapiens |
| PRRX1 paired related homeobox 1 Homo sapiens |
| PTGS2 prostaglandin-endoperoxide synthase 2 (prostaglandin G/H synthase and cyclooxygenase) Homo sapiens |
| PTPLA protein tyrosine phosphatase-like (proline instead of catalytic arginine), member A Homo sapiens |
| PTPRM protein tyrosine phosphatase, receptor type, M Homo sapiens |
| RFTN1 raftlin, lipid raft linker 1 Homo sapiens |
| SASH1 SAM and SH3 domain containing 1 Homo sapiens |
| SCG5 secretogranin V (7B2 protein) Homo sapiens |
| SEMA3C sema domain, immunoglobulin domain (Ig), short basic domain, secreted, (semaphorin) 3C Homo sapiens |
| SEMA6B sema domain, transmembrane domain (TM), and cytoplasmic domain, (semaphorin) 6B Homo sapiens |
| SEPT6 septin 6 Homo sapiens |
| SERPINA3 serpin peptidase inhibitor, clade A (alpha-1 antiproteinase, antitrypsin), member 3 Homo sapiens |
| SERPINF1 serpin peptidase inhibitor, clade F (alpha-2 antiplasmin, pigment epithelium derived factor), member 1 |
| SERTAD4 SERTA domain containing 4 Homo sapiens |
| SFRP1 secreted frizzled-related protein 1 Homo sapiens |
| SH3BGRL SH3 domain binding glutamic acid-rich protein like Homo sapiens |
| SH3PXD2A SH3 and PX domains 2A Homo sapiens |
| SHROOM2 shroom family member 2 Homo sapiens |
| SLC14A1 solute carrier family 14 (urea transporter), member 1 (Kidd blood group) Homo sapiens |
| SLC22A18 solute carrier family 22, member 18 Homo sapiens |
| SLC26A2 solute carrier family 26 (sulfate transporter), member 2 Homo sapiens |
| SLC6A10P solute carrier family 6 (neurotransmitter transporter, creatine), member 10 (pseudogene) |
| SNRPB2 small nuclear ribonucleoprotein polypeptide B'' Homo sapiens |
| SORBS2 sorbin and SH3 domain containing 2 Homo sapiens |
| SOX10 SRY (sex determining region Y)-box 10 Homo sapiens |
| SOX11 SRY (sex determining region Y)-box 11 Homo sapiens |
| SOX18 SRY (sex determining region Y)-box 18 Homo sapiens |
| SPAG1 sperm associated antigen 1 Homo sapiens |
| SPP1 secreted phosphoprotein 1 Homo sapiens |
| SPRY1 sprouty homolog 1, antagonist of FGF signaling (Drosophila) Homo sapiens |
| SPRY2 sprouty homolog 2 (Drosophila) Homo sapiens |
| SPRY4 sprouty homolog 4 (Drosophila) Homo sapiens |
| SPRYD5 SPRY domain containing 5 pseudogene; SPRY domain containing 5 Homo sapiens |
| ST3GAL5 ST3 beta-galactoside alpha-2,3-sialyltransferase 5 Homo sapiens |
| ST6GAL1 ST6 beta-galactosamide alpha-2,6-sialyltranferase 1 Homo sapiens |
| STC2 stanniocalcin 2 Homo sapiens |
| STMN3 stathmin-like 3 Homo sapiens |
| SYNM synemin, intermediate filament protein Homo sapiens |
| TAC1 tachykinin, precursor 1 Homo sapiens |
| TBC1D4 TBC1 domain family, member 4 Homo sapiens |
| TFPI tissue factor pathway inhibitor (lipoprotein-associated coagulation inhibitor) Homo sapiens |
| TGFA transforming growth factor, alpha Homo sapiens |
| TGFBI transforming growth factor, beta-induced, 68kDa Homo sapiens |
| TMEM132A transmembrane protein 132A Homo sapiens |
| TMEM154 transmembrane protein 154 Homo sapiens |
| TMEM45A transmembrane protein 45A Homo sapiens |
| TMEM47 transmembrane protein 47 Homo sapiens |
| TMTC2 transmembrane and tetratricopeptide repeat containing 2 Homo sapiens |
| TMTC4 transmembrane and tetratricopeptide repeat containing 4 Homo sapiens |
| TNFRSF11B tumor necrosis factor receptor superfamily, member 11b Homo sapiens |
| TNFRSF19 tumor necrosis factor receptor superfamily, member 19 Homo sapiens |
| TRIB2 tribbles homolog 2 (Drosophila) Homo sapiens |
| TRIML2 tripartite motif family-like 2 Homo sapiens |
| TRPC1 transient receptor potential cation channel, subfamily C, member 1 Homo sapiens |
| TSPAN33 tetraspanin 33 Homo sapiens |
| TSPAN5 tetraspanin 5 Homo sapiens |
| UGT8 UDP glycosyltransferase 8 Homo sapiens |
| VAMP8 vesicle-associated membrane protein 8 (endobrevin) Homo sapiens |
| VASN vasorin Homo sapiens |
| VAT1 vesicle amine transport protein 1 homolog (T. californica) Homo sapiens |
| VEGFC vascular endothelial growth factor C Homo sapiens |
| WWTR1 WW domain containing transcription regulator 1 Homo sapiens |
| ZCCHC24 zinc finger, CCHC domain containing 24 Homo sapiens |

**Supplementary Table 2. 852 differentially expressed common genes (816 annotated listed in the table) comparing A375 (BiR/MiR vs S) and SKMEL5 (BiR/MiR vs S). These genes define the resistance signature.**

|  | | | | | | |  |  |  |  |  |  |  |  |  |
| --- | --- | --- | --- | --- | --- | --- | --- | --- | --- | --- | --- | --- | --- | --- | --- |
| **OFFICIAL_GENE_SYMBOL Name Species** |  |  |  |  |  |  |  |  |  |  |  |  |  |  |  |
| \| ABCA3 ATP binding cassette subfamily A member 3(ABCA3) Homo sapiens \| \| \| \| \|  \|  \|  \| \| --- \| --- \| --- \| --- \| --- \| --- \| --- \| --- \| \| ABCB1 ATP binding cassette subfamily B member 1(ABCB1) Homo sapiens \| \| \| \| \|  \|  \|  \| \| ABCC3 ATP binding cassette subfamily C member 3(ABCC3) Homo sapiens \| \| \| \| \|  \|  \|  \| \| ABCC9 ATP binding cassette subfamily C member 9(ABCC9) Homo sapiens \| \| \| \| \|  \|  \|  \| \| ABCG2 ATP binding cassette subfamily G member 2 (Junior blood group)(ABCG2) Homo sapiens \| \| \| \| \| \| \|  \| \| ABI3 ABI family member 3(ABI3) Homo sapiens \| \|  \|  \|  \|  \|  \|  \| \| ABLIM1 actin binding LIM protein 1(ABLIM1) Homo sapiens \| \| \|  \|  \|  \|  \|  \| \| ACPL2 acid Phosphatase-Like Protein 2 Homo Sapiens \| \| \|  \|  \|  \|  \|  \| \| ACSL5 acyl-CoA synthetase long-chain family member 5(ACSL5) Homo sapiens \| \| \| \| \|  \|  \|  \| \| ACSM3 acyl-CoA synthetase medium-chain family member 3(ACSM3) Homo sapiens \| \| \| \| \|  \|  \|  \| \| ADAMTS17 ADAM metallopeptidase with thrombospondin type 1 motif 17(ADAMTS17) Homo sapiens \| \| \| \| \| \| \|  \| \| ADAMTSL1 ADAMTS like 1(ADAMTSL1) Homo sapiens \| \| \|  \|  \|  \|  \|  \| \| ADAMTSL4 ADAMTS like 4(ADAMTSL4) Homo sapiens \| \| \|  \|  \|  \|  \|  \| \| ADARB2 adenosine deaminase, RNA specific B2 (inactive)(ADARB2) Homo sapiens \| \| \| \| \|  \|  \|  \| \| ADCY1 adenylate cyclase 1(ADCY1) Homo sapiens \| \| \|  \|  \|  \|  \|  \| \| ADD2 adducin 2(ADD2) Homo sapiens \|  \|  \|  \|  \|  \|  \|  \| \| ADTRP androgen dependent TFPI regulating protein(ADTRP) Homo sapiens \| \| \| \| \|  \|  \|  \| \| AFP alpha fetoprotein(AFP) Homo sapiens \| \|  \|  \|  \|  \|  \|  \| \| AGRN agrin(AGRN) Homo sapiens \|  \|  \|  \|  \|  \|  \|  \| \| AIG1 androgen induced 1(AIG1) Homo sapiens \| \|  \|  \|  \|  \|  \|  \| \| ALCAM activated leukocyte cell adhesion molecule(ALCAM) Homo sapiens \| \| \| \| \|  \|  \|  \| \| ALDH2 aldehyde dehydrogenase 2 family (mitochondrial)(ALDH2) Homo sapiens \| \| \| \| \|  \|  \|  \| \| ALDH3B1 aldehyde dehydrogenase 3 family member B1(ALDH3B1) Homo sapiens \| \| \| \| \|  \|  \|  \| \| ALDOC aldolase, fructose-bisphosphate C(ALDOC) Homo sapiens \| \| \| \|  \|  \|  \|  \| \| ALOX5AP arachidonate 5-lipoxygenase activating protein(ALOX5AP) Homo sapiens \| \| \| \| \|  \|  \|  \| \| ALOXE3 arachidonate lipoxygenase 3(ALOXE3) Homo sapiens \| \| \| \|  \|  \|  \|  \| \| ALPK2 alpha kinase 2(ALPK2) Homo sapiens \| \|  \|  \|  \|  \|  \|  \| \| ALPP alkaline phosphatase, placental(ALPP) Homo sapiens \| \| \|  \|  \|  \|  \|  \| \| ALS2CL ALS2 C-terminal like(ALS2CL) Homo sapiens \| \| \|  \|  \|  \|  \|  \| \| AMPD3 adenosine monophosphate deaminase 3(AMPD3) Homo sapiens \| \| \| \| \|  \|  \|  \| \| ANKRD13A ankyrin repeat domain 13A(ANKRD13A) Homo sapiens \| \| \| \|  \|  \|  \|  \| \| ANO2 anoctamin 2(ANO2) Homo sapiens \| \|  \|  \|  \|  \|  \|  \| \| ANO4 anoctamin 4(ANO4) Homo sapiens \| \|  \|  \|  \|  \|  \|  \| \| ANXA8L2 annexin A8-Like Protein 2 Homo Sapiens \| \|  \|  \|  \|  \|  \|  \| \| AOAH acyloxyacyl hydrolase(AOAH) Homo sapiens \| \| \|  \|  \|  \|  \|  \| \| AOX1 aldehyde oxidase 1(AOX1) Homo sapiens \| \| \|  \|  \|  \|  \|  \| \| APLP1 amyloid beta precursor like protein 1(APLP1) Homo sapiens \| \| \| \|  \|  \|  \|  \| \| APOD apolipoprotein D(APOD) Homo sapiens \| \|  \|  \|  \|  \|  \|  \| \| APOL3 apolipoprotein L3(APOL3) Homo sapiens \| \| \|  \|  \|  \|  \|  \| \| APOL6 apolipoprotein L6(APOL6) Homo sapiens \| \| \|  \|  \|  \|  \|  \| \| AQP1 aquaporin 1 (Colton blood group)(AQP1) Homo sapiens \| \| \|  \|  \|  \|  \|  \| \| ARHGAP12 Rho GTPase activating protein 12(ARHGAP12) Homo sapiens \| \| \| \| \|  \|  \|  \| \| ARHGAP28 Rho GTPase activating protein 28(ARHGAP28) Homo sapiens \| \| \| \| \|  \|  \|  \| \| ARHGAP29 Rho GTPase activating protein 29(ARHGAP29) Homo sapiens \| \| \| \| \|  \|  \|  \| \| ARHGAP44 Rho GTPase activating protein 44(ARHGAP44) Homo sapiens \| \| \| \| \|  \|  \|  \| \| ARHGAP5-AS1 ARHGAP5 antisense RNA 1 (head to head)(ARHGAP5-AS1) Homo sapiens \| \| \| \| \| \|  \|  \| \| ARL14 ADP ribosylation factor like GTPase 14(ARL14) Homo sapiens \| \| \| \|  \|  \|  \|  \| \| ARMC4 armadillo repeat containing 4(ARMC4) Homo sapiens \| \| \|  \|  \|  \|  \|  \| \| ARNT2 aryl hydrocarbon receptor nuclear translocator 2(ARNT2) Homo sapiens \| \| \| \| \|  \|  \|  \| \| ARNTL2 aryl hydrocarbon receptor nuclear translocator like 2(ARNTL2) Homo sapiens \| \| \| \| \| \|  \|  \| \| ARPP21 cAMP regulated phosphoprotein 21(ARPP21) Homo sapiens \| \| \| \|  \|  \|  \|  \| \| ART3 ADP-ribosyltransferase 3(ART3) Homo sapiens \| \| \|  \|  \|  \|  \|  \| \| ASS1 argininosuccinate synthase 1(ASS1) Homo sapiens \| \| \|  \|  \|  \|  \|  \| \| ASTN1 astrotactin 1(ASTN1) Homo sapiens \| \|  \|  \|  \|  \|  \|  \| \| ATF3 activating transcription factor 3(ATF3) Homo sapiens \| \| \|  \|  \|  \|  \|  \| \| ATP2B4 ATPase plasma membrane Ca2+ transporting 4(ATP2B4) Homo sapiens \| \| \| \| \|  \|  \|  \| \| ATP8B3 ATPase phospholipid transporting 8B3(ATP8B3) Homo sapiens \| \| \| \|  \|  \|  \|  \| \| B3GALT2 beta-1,3-galactosyltransferase 2(B3GALT2) Homo sapiens \| \| \| \|  \|  \|  \|  \| \| B3GAT1 beta-1,3-glucuronyltransferase 1(B3GAT1) Homo sapiens \| \| \| \|  \|  \|  \|  \| \| B3GNT4 UDP-GlcNAc:betaGal beta-1,3-N-acetylglucosaminyltransferase 4(B3GNT4) Homo sapiens \| \| \| \| \| \| \|  \| \| B4GALT1 beta-1,4-galactosyltransferase 1(B4GALT1) Homo sapiens \| \| \| \|  \|  \|  \|  \| \| B4GALT6 beta-1,4-galactosyltransferase 6(B4GALT6) Homo sapiens \| \| \| \|  \|  \|  \|  \| \| BAI2 brain-Specific Angiogenesis Inhibitor 2 Homo Sapiens \| \| \|  \|  \|  \|  \|  \| \| BARX1 BARX homeobox 1(BARX1) Homo sapiens \| \|  \|  \|  \|  \|  \|  \| \| BASP1 brain abundant membrane attached signal protein 1(BASP1) Homo sapiens \| \| \| \| \| \|  \|  \| \| BCL6 B-cell CLL/lymphoma 6(BCL6) Homo sapiens \| \| \|  \|  \|  \|  \|  \| \| BDKRB1 bradykinin receptor B1(BDKRB1) Homo sapiens \| \| \|  \|  \|  \|  \|  \| \| BEGAIN brain enriched guanylate kinase associated(BEGAIN) Homo sapiens \| \| \| \| \|  \|  \|  \| \| BEX1 brain expressed X-linked 1(BEX1) Homo sapiens \| \| \|  \|  \|  \|  \|  \| \| BGN biglycan(BGN) Homo sapiens \|  \|  \|  \|  \|  \|  \|  \| \| BHLHE40 basic helix-loop-helix family member e40(BHLHE40) Homo sapiens \| \| \| \| \|  \|  \|  \| \| BIN3 bridging integrator 3(BIN3) Homo sapiens \| \|  \|  \|  \|  \|  \|  \| \| BMF Bcl2 modifying factor(BMF) Homo sapiens \| \|  \|  \|  \|  \|  \|  \| \| BMP4 bone morphogenetic protein 4(BMP4) Homo sapiens \| \| \|  \|  \|  \|  \|  \| \| BMP8B bone morphogenetic protein 8b(BMP8B) Homo sapiens \| \| \| \|  \|  \|  \|  \| \| BNIP3 BCL2 interacting protein 3(BNIP3) Homo sapiens \| \| \|  \|  \|  \|  \|  \| \| BNIP3L BCL2 interacting protein 3 like(BNIP3L) Homo sapiens \| \| \|  \|  \|  \|  \|  \| \| BST2 bone marrow stromal cell antigen 2(BST2) Homo sapiens \| \| \| \|  \|  \|  \|  \| \| C10orf90 chromosome 10 open reading frame 90(C10orf90) Homo sapiens \| \| \| \| \|  \|  \|  \| \| C11orf45 chromosome 11 open reading frame 45(C11orf45) Homo sapiens \| \| \| \| \|  \|  \|  \| \| C14orf132 chromosome 14 open reading frame 132(C14orf132) Homo sapiens \| \| \| \| \|  \|  \|  \| \| C15orf48 chromosome 15 open reading frame 48(C15orf48) Homo sapiens \| \| \| \| \|  \|  \|  \| \| C16orf52 chromosome 16 open reading frame 52(C16orf52) Homo sapiens \| \| \| \| \|  \|  \|  \| \| C16orf74 chromosome 16 open reading frame 74(C16orf74) Homo sapiens \| \| \| \| \|  \|  \|  \| \| C17orf97 chromosome 17 open reading frame 97(C17orf97) Homo sapiens \| \| \| \| \|  \|  \|  \| \| C19orf33 chromosome 19 open reading frame 33(C19orf33) Homo sapiens \| \| \| \| \|  \|  \|  \| \| C1orf54 chromosome 1 open reading frame 54(C1orf54) Homo sapiens \| \| \| \|  \|  \|  \|  \| \| C1R complement C1r(C1R) Homo sapiens \| \|  \|  \|  \|  \|  \|  \| \| C1S complement C1s(C1S) Homo sapiens \| \|  \|  \|  \|  \|  \|  \| \| C20orf141 chromosome 20 open reading frame 141(C20orf141) Homo sapiens \| \| \| \| \|  \|  \|  \| \| C3 complement C3(C3) Homo sapiens \| \|  \|  \|  \|  \|  \|  \| \| C5orf56 chromosome 5 open reading frame 56(C5orf56) Homo sapiens \| \| \| \|  \|  \|  \|  \| \| C5orf58 chromosome 5 open reading frame 58(C5orf58) Homo sapiens \| \| \| \|  \|  \|  \|  \| \| C9orf84 chromosome 9 open reading frame 84(C9orf84) Homo sapiens \| \| \| \|  \|  \|  \|  \| \| CA11 carbonic anhydrase 11(CA11) Homo sapiens \| \| \|  \|  \|  \|  \|  \| \| CA9 carbonic anhydrase 9(CA9) Homo sapiens \| \|  \|  \|  \|  \|  \|  \| \| CACNA1C calcium voltage-gated channel subunit alpha1 C(CACNA1C) Homo sapiens \| \| \| \| \|  \|  \|  \| \| CADM4 cell adhesion molecule 4(CADM4) Homo sapiens \| \| \|  \|  \|  \|  \|  \| \| CAMK1D calcium/calmodulin dependent protein kinase ID(CAMK1D) Homo sapiens \| \| \| \| \|  \|  \|  \| \| CAP2 CAP, adenylate cyclase-associated protein, 2 (yeast)(CAP2) Homo sapiens \| \| \| \| \| \|  \|  \| \| CAPS calcyphosine(CAPS) Homo sapiens \| \|  \|  \|  \|  \|  \|  \| \| CAPSL calcyphosine like(CAPSL) Homo sapiens \| \| \|  \|  \|  \|  \|  \| \| CARD11 caspase recruitment domain family member 11(CARD11) Homo sapiens \| \| \| \| \|  \|  \|  \| \| CASP1 caspase 1(CASP1) Homo sapiens \| \|  \|  \|  \|  \|  \|  \| \| CASP6 caspase 6(CASP6) Homo sapiens \| \|  \|  \|  \|  \|  \|  \| \| CASQ1 calsequestrin 1(CASQ1) Homo sapiens \| \|  \|  \|  \|  \|  \|  \| \| CASZ1 castor zinc finger 1(CASZ1) Homo sapiens \| \| \|  \|  \|  \|  \|  \| \| CCBE1 collagen and calcium binding EGF domains 1(CCBE1) Homo sapiens \| \| \| \| \|  \|  \|  \| \| CCDC147 coiled-Coil Domain-Containing 147 Homo Sapiens \| \| \|  \|  \|  \|  \|  \| \| CCDC102B coiled-coil domain containing 102B(CCDC102B) Homo sapiens \| \| \| \| \|  \|  \|  \| \| CCDC80 coiled-coil domain containing 80(CCDC80) Homo sapiens \| \| \| \|  \|  \|  \|  \| \| CCDC85A coiled-coil domain containing 85A(CCDC85A) Homo sapiens \| \| \| \|  \|  \|  \|  \| \| CCDC85C coiled-coil domain containing 85C(CCDC85C) Homo sapiens \| \| \| \|  \|  \|  \|  \| \| CCDC92 coiled-coil domain containing 92(CCDC92) Homo sapiens \| \| \| \|  \|  \|  \|  \| \| CCL2 C-C motif chemokine ligand 2(CCL2) Homo sapiens \| \| \|  \|  \|  \|  \|  \| \| CCL26 C-C motif chemokine ligand 26(CCL26) Homo sapiens \| \| \|  \|  \|  \|  \|  \| \| CCL3 C-C motif chemokine ligand 3(CCL3) Homo sapiens \| \| \|  \|  \|  \|  \|  \| \| CCL4 C-C motif chemokine ligand 4(CCL4) Homo sapiens \| \| \|  \|  \|  \|  \|  \| \| CCL5 C-C motif chemokine ligand 5(CCL5) Homo sapiens \| \| \|  \|  \|  \|  \|  \| \| CD24 CD24 molecule(CD24) Homo sapiens \| \|  \|  \|  \|  \|  \|  \| \| CD274 CD274 molecule(CD274) Homo sapiens \| \|  \|  \|  \|  \|  \|  \| \| CD68 CD68 molecule(CD68) Homo sapiens \| \|  \|  \|  \|  \|  \|  \| \| CD74 CD74 molecule(CD74) Homo sapiens \| \|  \|  \|  \|  \|  \|  \| \| CD82 CD82 molecule(CD82) Homo sapiens \| \|  \|  \|  \|  \|  \|  \| \| CD96 CD96 molecule(CD96) Homo sapiens \| \|  \|  \|  \|  \|  \|  \| \| CDA cytidine deaminase(CDA) Homo sapiens \| \|  \|  \|  \|  \|  \|  \| \| CDC42EP1 CDC42 effector protein 1(CDC42EP1) Homo sapiens \| \| \| \|  \|  \|  \|  \| \| CDH10 cadherin 10(CDH10) Homo sapiens \| \|  \|  \|  \|  \|  \|  \| \| CDH18 cadherin 18(CDH18) Homo sapiens \| \|  \|  \|  \|  \|  \|  \| \| CDK15 cyclin dependent kinase 15(CDK15) Homo sapiens \| \| \|  \|  \|  \|  \|  \| \| CDK2AP1 cyclin dependent kinase 2 associated protein 1(CDK2AP1) Homo sapiens \| \| \| \| \|  \|  \|  \| \| CDKN1C cyclin dependent kinase inhibitor 1C(CDKN1C) Homo sapiens \| \| \| \|  \|  \|  \|  \| \| CDR2L cerebellar degeneration related protein 2 like(CDR2L) Homo sapiens \| \| \| \| \|  \|  \|  \| \| CDS1 CDP-diacylglycerol synthase 1(CDS1) Homo sapiens \| \| \|  \|  \|  \|  \|  \| \| CEP85L centrosomal protein 85 like(CEP85L) Homo sapiens \| \| \|  \|  \|  \|  \|  \| \| CERCAM cerebral endothelial cell adhesion molecule(CERCAM) Homo sapiens \| \| \| \| \|  \|  \|  \| \| CFB complement factor B(CFB) Homo sapiens \| \| \|  \|  \|  \|  \|  \| \| CFH complement factor H(CFH) Homo sapiens \| \| \|  \|  \|  \|  \|  \| \| CGB5 chorionic gonadotropin beta subunit 5(CGB5) Homo sapiens \| \| \| \|  \|  \|  \|  \| \| CGB8 chorionic gonadotropin beta subunit 8(CGB8) Homo sapiens \| \| \| \|  \|  \|  \|  \| \| CGNL1 cingulin like 1(CGNL1) Homo sapiens \| \|  \|  \|  \|  \|  \|  \| \| CHMP4C charged multivesicular body protein 4C(CHMP4C) Homo sapiens \| \| \| \|  \|  \|  \|  \| \| CHST4 carbohydrate sulfotransferase 4(CHST4) Homo sapiens \| \| \|  \|  \|  \|  \|  \| \| CHST6 carbohydrate sulfotransferase 6(CHST6) Homo sapiens \| \| \|  \|  \|  \|  \|  \| \| CHSY1 chondroitin sulfate synthase 1(CHSY1) Homo sapiens \| \| \|  \|  \|  \|  \|  \| \| CIITA class II major histocompatibility complex transactivator(CIITA) Homo sapiens \| \| \| \| \|  \|  \|  \| \| CITED2 Cbp/p300 interacting transactivator with Glu/Asp rich carboxy-terminal domain 2(CITED2) Homo sapiens \| \| \| \| \| \| \| \| \| CLDN14 claudin 14(CLDN14) Homo sapiens \| \|  \|  \|  \|  \|  \|  \| \| CLIC3 chloride intracellular channel 3(CLIC3) Homo sapiens \| \| \|  \|  \|  \|  \|  \| \| CLMN calmin(CLMN) Homo sapiens \|  \|  \|  \|  \|  \|  \|  \| \| CLMP CXADR like membrane protein(CLMP) Homo sapiens \| \| \| \|  \|  \|  \|  \| \| CLU clusterin(CLU) Homo sapiens \|  \|  \|  \|  \|  \|  \|  \| \| CNFN cornifelin(CNFN) Homo sapiens \| \|  \|  \|  \|  \|  \|  \| \| CNTN1 contactin 1(CNTN1) Homo sapiens \| \|  \|  \|  \|  \|  \|  \| \| COL17A1 collagen type XVII alpha 1 chain(COL17A1) Homo sapiens \| \| \| \|  \|  \|  \|  \| \| COL19A1 collagen type XIX alpha 1 chain(COL19A1) Homo sapiens \| \| \| \|  \|  \|  \|  \| \| COL1A1 collagen type I alpha 1 chain(COL1A1) Homo sapiens \| \| \|  \|  \|  \|  \|  \| \| COL3A1 collagen type III alpha 1 chain(COL3A1) Homo sapiens \| \| \|  \|  \|  \|  \|  \| \| COL4A5 collagen type IV alpha 5 chain(COL4A5) Homo sapiens \| \| \|  \|  \|  \|  \|  \| \| COL5A1 collagen type V alpha 1 chain(COL5A1) Homo sapiens \| \| \| \|  \|  \|  \|  \| \| COL9A3 collagen type IX alpha 3 chain(COL9A3) Homo sapiens \| \| \|  \|  \|  \|  \|  \| \| COPZ2 coatomer protein complex subunit zeta 2(COPZ2) Homo sapiens \| \| \| \|  \|  \|  \|  \| \| CPA4 carboxypeptidase A4(CPA4) Homo sapiens \| \| \|  \|  \|  \|  \|  \| \| CPN1 carboxypeptidase N subunit 1(CPN1) Homo sapiens \| \| \|  \|  \|  \|  \|  \| \| CPZ carboxypeptidase Z(CPZ) Homo sapiens \| \|  \|  \|  \|  \|  \|  \| \| CREB3L1 cAMP responsive element binding protein 3 like 1(CREB3L1) Homo sapiens \| \| \| \| \| \|  \|  \| \| CRISPLD2 cysteine rich secretory protein LCCL domain containing 2(CRISPLD2) Homo sapiens \| \| \| \| \| \| \|  \| \| CSDC2 cold shock domain containing C2(CSDC2) Homo sapiens \| \| \| \|  \|  \|  \|  \| \| CSF1R colony stimulating factor 1 receptor(CSF1R) Homo sapiens \| \| \| \|  \|  \|  \|  \| \| CSPG5 chondroitin sulfate proteoglycan 5(CSPG5) Homo sapiens \| \| \| \|  \|  \|  \|  \| \| CTH cystathionine gamma-lyase(CTH) Homo sapiens \| \| \|  \|  \|  \|  \|  \| \| CTSS cathepsin S(CTSS) Homo sapiens \| \|  \|  \|  \|  \|  \|  \| \| CXADR coxsackie virus and adenovirus receptor(CXADR) Homo sapiens \| \| \| \|  \|  \|  \|  \| \| CXCL10 C-X-C motif chemokine ligand 10(CXCL10) Homo sapiens \| \| \| \|  \|  \|  \|  \| \| CXCL11 C-X-C motif chemokine ligand 11(CXCL11) Homo sapiens \| \| \| \|  \|  \|  \|  \| \| CXCR7 C-X-C Chemokine Receptor Type 7 Homo Sapiens \| \| \|  \|  \|  \|  \|  \| \| CYBA cytochrome b-245 alpha chain(CYBA) Homo sapiens \| \| \|  \|  \|  \|  \|  \| \| CYCSP52 cytochrome c, somatic pseudogene 52(CYCSP52) Homo sapiens \| \| \| \| \|  \|  \|  \| \| CYP11A1 cytochrome P450 family 11 subfamily A member 1(CYP11A1) Homo sapiens \| \| \| \| \|  \|  \|  \| \| CYP1B1 cytochrome P450 family 1 subfamily B member 1(CYP1B1) Homo sapiens \| \| \| \| \|  \|  \|  \| \| CYP26B1 cytochrome P450 family 26 subfamily B member 1(CYP26B1) Homo sapiens \| \| \| \| \|  \|  \|  \| \| CYR61 cysteine rich angiogenic inducer 61(CYR61) Homo sapiens \| \| \| \|  \|  \|  \|  \| \| CYTIP cytohesin 1 interacting protein(CYTIP) Homo sapiens \| \| \|  \|  \|  \|  \|  \| \| DCBLD1 discoidin, CUB and LCCL domain containing 1(DCBLD1) Homo sapiens \| \| \| \| \|  \|  \|  \| \| DCC DCC netrin 1 receptor(DCC) Homo sapiens \| \|  \|  \|  \|  \|  \|  \| \| DCLK2 doublecortin like kinase 2(DCLK2) Homo sapiens \| \| \|  \|  \|  \|  \|  \| \| DDX58 DExD/H-box helicase 58(DDX58) Homo sapiens \| \| \|  \|  \|  \|  \|  \| \| DDX60 DExD/H-box helicase 60(DDX60) Homo sapiens \| \| \|  \|  \|  \|  \|  \| \| DDX60L DEAD-box helicase 60-like(DDX60L) Homo sapiens \| \| \|  \|  \|  \|  \|  \| \| DENND2A DENN domain containing 2A(DENND2A) Homo sapiens \| \| \| \|  \|  \|  \|  \| \| DGCR9 DiGeorge syndrome critical region gene 9 (non-protein coding)(DGCR9) Homo sapiens \| \| \| \| \| \|  \|  \| \| DGKD diacylglycerol kinase delta(DGKD) Homo sapiens \| \| \|  \|  \|  \|  \|  \| \| DIO2 deiodinase, iodothyronine type II(DIO2) Homo sapiens \| \| \|  \|  \|  \|  \|  \| \| DKK3 dickkopf WNT signaling pathway inhibitor 3(DKK3) Homo sapiens \| \| \| \| \|  \|  \|  \| \| DLX4 distal-less homeobox 4(DLX4) Homo sapiens \| \| \|  \|  \|  \|  \|  \| \| DMKN dermokine(DMKN) Homo sapiens \|  \|  \|  \|  \|  \|  \|  \| \| DNAH9 dynein axonemal heavy chain 9(DNAH9) Homo sapiens \| \| \| \|  \|  \|  \|  \| \| DNAJB2 DnaJ heat shock protein family (Hsp40) member B2(DNAJB2) Homo sapiens \| \| \| \| \| \|  \|  \| \| DNAJC12 DnaJ heat shock protein family (Hsp40) member C12(DNAJC12) Homo sapiens \| \| \| \| \| \|  \|  \| \| DNASE1L2 deoxyribonuclease 1 like 2(DNASE1L2) Homo sapiens \| \| \| \|  \|  \|  \|  \| \| DOCK2 dedicator of cytokinesis 2(DOCK2) Homo sapiens \| \| \|  \|  \|  \|  \|  \| \| DOCK4 dedicator of cytokinesis 4(DOCK4) Homo sapiens \| \| \|  \|  \|  \|  \|  \| \| DSE dermatan sulfate epimerase(DSE) Homo sapiens \| \| \|  \|  \|  \|  \|  \| \| DSEL dermatan sulfate epimerase-like(DSEL) Homo sapiens \| \| \|  \|  \|  \|  \|  \| \| DSP desmoplakin(DSP) Homo sapiens \| \|  \|  \|  \|  \|  \|  \| \| DTNA dystrobrevin alpha(DTNA) Homo sapiens \| \|  \|  \|  \|  \|  \|  \| \| DUSP27 dual specificity phosphatase 27 (putative)(DUSP27) Homo sapiens \| \| \| \| \|  \|  \|  \| \| DUSP4 dual specificity phosphatase 4(DUSP4) Homo sapiens \| \| \|  \|  \|  \|  \|  \| \| DUSP6 dual specificity phosphatase 6(DUSP6) Homo sapiens \| \| \|  \|  \|  \|  \|  \| \| DUSP8 dual specificity phosphatase 8(DUSP8) Homo sapiens \| \| \|  \|  \|  \|  \|  \| \| DYSF dysferlin(DYSF) Homo sapiens \|  \|  \|  \|  \|  \|  \|  \| \| EBI3 Epstein-Barr virus induced 3(EBI3) Homo sapiens \| \| \|  \|  \|  \|  \|  \| \| EDN1 endothelin 1(EDN1) Homo sapiens \| \|  \|  \|  \|  \|  \|  \| \| EDN2 endothelin 2(EDN2) Homo sapiens \| \|  \|  \|  \|  \|  \|  \| \| EEF1A2 eukaryotic translation elongation factor 1 alpha 2(EEF1A2) Homo sapiens \| \| \| \| \|  \|  \|  \| \| EFEMP2 EGF containing fibulin like extracellular matrix protein 2(EFEMP2) Homo sapiens \| \| \| \| \| \|  \|  \| \| EGLN3 egl-9 family hypoxia inducible factor 3(EGLN3) Homo sapiens \| \| \| \|  \|  \|  \|  \| \| EGR1 early growth response 1(EGR1) Homo sapiens \| \| \|  \|  \|  \|  \|  \| \| EGR2 early growth response 2(EGR2) Homo sapiens \| \| \|  \|  \|  \|  \|  \| \| EGR3 early growth response 3(EGR3) Homo sapiens \| \| \|  \|  \|  \|  \|  \| \| EHD2 EH domain containing 2(EHD2) Homo sapiens \| \| \|  \|  \|  \|  \|  \| \| EIF4EBP3 eukaryotic translation initiation factor 4E binding protein 3(EIF4EBP3) Homo sapiens \| \| \| \| \| \| \|  \| \| ELFN2 extracellular leucine rich repeat and fibronectin type III domain containing 2(ELFN2) Homo sapiens \| \| \| \| \| \| \|  \| \| ELL2 elongation factor for RNA polymerase II 2(ELL2) Homo sapiens \| \| \| \|  \|  \|  \|  \| \| ELOVL7 ELOVL fatty acid elongase 7(ELOVL7) Homo sapiens \| \| \| \|  \|  \|  \|  \| \| EMP1 epithelial membrane protein 1(EMP1) Homo sapiens \| \| \|  \|  \|  \|  \|  \| \| EN2 engrailed homeobox 2(EN2) Homo sapiens \| \| \|  \|  \|  \|  \|  \| \| ENC1 ectodermal-neural cortex 1(ENC1) Homo sapiens \| \| \|  \|  \|  \|  \|  \| \| EPHA5 EPH receptor A5(EPHA5) Homo sapiens \| \|  \|  \|  \|  \|  \|  \| \| EPHB2 EPH receptor B2(EPHB2) Homo sapiens \| \|  \|  \|  \|  \|  \|  \| \| EPHB3 EPH receptor B3(EPHB3) Homo sapiens \| \|  \|  \|  \|  \|  \|  \| \| EPS8L2 EPS8 like 2(EPS8L2) Homo sapiens \| \|  \|  \|  \|  \|  \|  \| \| ERBB3 erb-b2 receptor tyrosine kinase 3(ERBB3) Homo sapiens \| \| \| \|  \|  \|  \|  \| \| ERRFI1 ERBB receptor feedback inhibitor 1(ERRFI1) Homo sapiens \| \| \| \|  \|  \|  \|  \| \| ETV1 ETS variant 1(ETV1) Homo sapiens \| \|  \|  \|  \|  \|  \|  \| \| ETV4 ETS variant 4(ETV4) Homo sapiens \| \|  \|  \|  \|  \|  \|  \| \| EXTL1 exostosin like glycosyltransferase 1(EXTL1) Homo sapiens \| \| \| \|  \|  \|  \|  \| \| EYA1 EYA transcriptional coactivator and phosphatase 1(EYA1) Homo sapiens \| \| \| \| \|  \|  \|  \| \| F11R F11 receptor(F11R) Homo sapiens \| \|  \|  \|  \|  \|  \|  \| \| FABP3 fatty acid binding protein 3(FABP3) Homo sapiens \| \| \|  \|  \|  \|  \|  \| \| FABP7 fatty acid binding protein 7(FABP7) Homo sapiens \| \| \|  \|  \|  \|  \|  \| \| FAM127B family with sequence similarity 127 member B(FAM127B) Homo sapiens \| \| \| \| \|  \|  \|  \| \| FAM162A family with sequence similarity 162 member A(FAM162A) Homo sapiens \| \| \| \| \|  \|  \|  \| \| FAM167A family with sequence similarity 167 member A(FAM167A) Homo sapiens \| \| \| \| \|  \|  \|  \| \| FAM19A3 family with sequence similarity 19 member A3, C-C motif chemokine like(FAM19A3) Homo sapiens \| \| \| \| \| \| \| \| \| FAM25A family with sequence similarity 25 member A(FAM25A) Homo sapiens \| \| \| \| \|  \|  \|  \| \| FAM26E family with sequence similarity 26 member E(FAM26E) Homo sapiens \| \| \| \| \|  \|  \|  \| \| FAM49A family with sequence similarity 49 member A(FAM49A) Homo sapiens \| \| \| \| \|  \|  \|  \| \| FAM83A family with sequence similarity 83 member A(FAM83A) Homo sapiens \| \| \| \| \|  \|  \|  \| \| FARP1 FERM, ARH/RhoGEF and pleckstrin domain protein 1(FARP1) Homo sapiens \| \| \| \| \|  \|  \|  \| \| FAT4 FAT atypical cadherin 4(FAT4) Homo sapiens \| \| \|  \|  \|  \|  \|  \| \| FBN1 fibrillin 1(FBN1) Homo sapiens \|  \|  \|  \|  \|  \|  \|  \| \| FBXL7 F-box and leucine rich repeat protein 7(FBXL7) Homo sapiens \| \| \| \|  \|  \|  \|  \| \| FBXO2 F-box protein 2(FBXO2) Homo sapiens \| \|  \|  \|  \|  \|  \|  \| \| FDCSP follicular dendritic cell secreted protein(FDCSP) Homo sapiens \| \| \| \|  \|  \|  \|  \| \| FER1L4 fer-1 like family member 4, pseudogene(FER1L4) Homo sapiens \| \| \| \|  \|  \|  \|  \| \| FGF11 fibroblast growth factor 11(FGF11) Homo sapiens \| \| \|  \|  \|  \|  \|  \| \| FGF13 fibroblast growth factor 13(FGF13) Homo sapiens \| \| \|  \|  \|  \|  \|  \| \| FGF2 fibroblast growth factor 2(FGF2) Homo sapiens \| \| \|  \|  \|  \|  \|  \| \| FGFBP1 fibroblast growth factor binding protein 1(FGFBP1) Homo sapiens \| \| \| \| \|  \|  \|  \| \| FGFBP2 fibroblast growth factor binding protein 2(FGFBP2) Homo sapiens \| \| \| \| \|  \|  \|  \| \| FHL1 four and a half LIM domains 1(FHL1) Homo sapiens \| \| \|  \|  \|  \|  \|  \| \| FIBCD1 fibrinogen C domain containing 1(FIBCD1) Homo sapiens \| \| \| \|  \|  \|  \|  \| \| FLJ20021 uncharacterized LOC90024(FLJ20021) Homo sapiens \| \| \| \|  \|  \|  \|  \| \| FLJ22447 uncharacterized LOC400221(FLJ22447) Homo sapiens \| \| \| \|  \|  \|  \|  \| \| FLJ23867 uncharacterized protein FLJ23867(FLJ23867) Homo sapiens \| \| \| \| \|  \|  \|  \| \| FLNB filamin B(FLNB) Homo sapiens \|  \|  \|  \|  \|  \|  \|  \| \| FLRT3 fibronectin leucine rich transmembrane protein 3(FLRT3) Homo sapiens \| \| \| \| \|  \|  \|  \| \| FLT1 fms related tyrosine kinase 1(FLT1) Homo sapiens \| \| \|  \|  \|  \|  \|  \| \| FMNL3 formin like 3(FMNL3) Homo sapiens \| \|  \|  \|  \|  \|  \|  \| \| FNDC4 fibronectin type III domain containing 4(FNDC4) Homo sapiens \| \| \| \|  \|  \|  \|  \| \| FOLR1 folate receptor 1(FOLR1) Homo sapiens \| \|  \|  \|  \|  \|  \|  \| \| FOS Fos proto-oncogene, AP-1 transcription factor subunit(FOS) Homo sapiens \| \| \| \| \|  \|  \|  \| \| FOSB FosB proto-oncogene, AP-1 transcription factor subunit(FOSB) Homo sapiens \| \| \| \| \| \|  \|  \| \| FOSL2 FOS like 2, AP-1 transcription factor subunit(FOSL2) Homo sapiens \| \| \| \| \|  \|  \|  \| \| FOXD2 forkhead box D2(FOXD2) Homo sapiens \| \|  \|  \|  \|  \|  \|  \| \| FOXP1 forkhead box P1(FOXP1) Homo sapiens \| \|  \|  \|  \|  \|  \|  \| \| FPR1 formyl peptide receptor 1(FPR1) Homo sapiens \| \| \|  \|  \|  \|  \|  \| \| FRAS1 Fraser extracellular matrix complex subunit 1(FRAS1) Homo sapiens \| \| \| \| \|  \|  \|  \| \| FREM1 FRAS1 related extracellular matrix 1(FREM1) Homo sapiens \| \| \| \|  \|  \|  \|  \| \| FREM2 FRAS1 related extracellular matrix protein 2(FREM2) Homo sapiens \| \| \| \| \|  \|  \|  \| \| FRMD4B FERM domain containing 4B(FRMD4B) Homo sapiens \| \| \| \|  \|  \|  \|  \| \| FRMD8 FERM domain containing 8(FRMD8) Homo sapiens \| \| \|  \|  \|  \|  \|  \| \| FSTL1 follistatin like 1(FSTL1) Homo sapiens \| \|  \|  \|  \|  \|  \|  \| \| FSTL3 follistatin like 3(FSTL3) Homo sapiens \| \|  \|  \|  \|  \|  \|  \| \| FTH1 ferritin heavy chain 1(FTH1) Homo sapiens \| \|  \|  \|  \|  \|  \|  \| \| FXYD3 FXYD domain containing ion transport regulator 3(FXYD3) Homo sapiens \| \| \| \| \|  \|  \|  \| \| FZD1 frizzled class receptor 1(FZD1) Homo sapiens \| \|  \|  \|  \|  \|  \|  \| \| FZD8 frizzled class receptor 8(FZD8) Homo sapiens \| \|  \|  \|  \|  \|  \|  \| \| GABRE gamma-aminobutyric acid type A receptor epsilon subunit(GABRE) Homo sapiens \| \| \| \| \| \|  \|  \| \| GALNT10 polypeptide N-acetylgalactosaminyltransferase 10(GALNT10) Homo sapiens \| \| \| \| \| \|  \|  \| \| GAP43 growth associated protein 43(GAP43) Homo sapiens \| \| \|  \|  \|  \|  \|  \| \| GAS6 growth arrest specific 6(GAS6) Homo sapiens \| \| \|  \|  \|  \|  \|  \| \| GAS7 growth arrest specific 7(GAS7) Homo sapiens \| \| \|  \|  \|  \|  \|  \| \| GCNT1 glucosaminyl (N-acetyl) transferase 1, core 2(GCNT1) Homo sapiens \| \| \| \| \|  \|  \|  \| \| GDF15 growth differentiation factor 15(GDF15) Homo sapiens \| \| \|  \|  \|  \|  \|  \| \| GDNF glial cell derived neurotrophic factor(GDNF) Homo sapiens \| \| \| \|  \|  \|  \|  \| \| GDPD3 glycerophosphodiester phosphodiesterase domain containing 3(GDPD3) Homo sapiens \| \| \| \| \| \| \|  \| \| GDPD5 glycerophosphodiester phosphodiesterase domain containing 5(GDPD5) Homo sapiens \| \| \| \| \| \| \|  \| \| GFRA1 GDNF family receptor alpha 1(GFRA1) Homo sapiens \| \| \| \|  \|  \|  \|  \| \| GGT1 gamma-glutamyltransferase 1(GGT1) Homo sapiens \| \| \|  \|  \|  \|  \|  \| \| GGT8P gamma-glutamyltransferase 8 pseudogene(GGT8P) Homo sapiens \| \| \| \| \|  \|  \|  \| \| GIMAP5 GTPase, IMAP family member 5(GIMAP5) Homo sapiens \| \| \| \|  \|  \|  \|  \| \| GJB1 gap junction protein beta 1(GJB1) Homo sapiens \| \| \|  \|  \|  \|  \|  \| \| GLDC glycine decarboxylase(GLDC) Homo sapiens \| \| \|  \|  \|  \|  \|  \| \| GLIPR1 GLI pathogenesis related 1(GLIPR1) Homo sapiens \| \| \|  \|  \|  \|  \|  \| \| GLIPR2 GLI pathogenesis related 2(GLIPR2) Homo sapiens \| \| \|  \|  \|  \|  \|  \| \| GLIS1 GLIS family zinc finger 1(GLIS1) Homo sapiens \| \| \|  \|  \|  \|  \|  \| \| GLP2R glucagon like peptide 2 receptor(GLP2R) Homo sapiens \| \| \|  \|  \|  \|  \|  \| \| GNAI1 G protein subunit alpha i1(GNAI1) Homo sapiens \| \| \|  \|  \|  \|  \|  \| \| GNAO1 G protein subunit alpha o1(GNAO1) Homo sapiens \| \| \|  \|  \|  \|  \|  \| \| GNGT2 G protein subunit gamma transducin 2(GNGT2) Homo sapiens \| \| \| \|  \|  \|  \|  \| \| GPER G Protein-Coupled Estrogen Receptor 1 Homo Sapiens \| \| \| \|  \|  \|  \|  \| \| GPR1 G protein-coupled receptor 1(GPR1) Homo sapiens \| \| \|  \|  \|  \|  \|  \| \| GPR115 G protein-coupled receptor 115(GPR115) Homo sapiens \| \| \| \|  \|  \|  \|  \| \| GPR153 G protein-coupled receptor 153(GPR153) Homo sapiens \| \| \| \|  \|  \|  \|  \| \| GPR3 G protein-coupled receptor 3(GPR3) Homo sapiens \| \| \|  \|  \|  \|  \|  \| \| GPRASP2 G protein-coupled receptor associated sorting protein 2(GPRASP2) Homo sapiens \| \| \| \| \| \|  \|  \| \| GPRC5C G protein-coupled receptor class C group 5 member C(GPRC5C) Homo sapiens \| \| \| \| \| \|  \|  \| \| GRAMD3 GRAM domain containing 3(GRAMD3) Homo sapiens \| \| \| \|  \|  \|  \|  \| \| GRASP general receptor for phosphoinositides 1 associated scaffold protein(GRASP) Homo sapiens \| \| \| \| \| \| \|  \| \| GRB14 growth factor receptor bound protein 14(GRB14) Homo sapiens \| \| \| \|  \|  \|  \|  \| \| GRIK2 glutamate ionotropic receptor kainate type subunit 2(GRIK2) Homo sapiens \| \| \| \| \|  \|  \|  \| \| HAND2 heart and neural crest derivatives expressed 2(HAND2) Homo sapiens \| \| \| \| \|  \|  \|  \| \| HAPLN3 hyaluronan and proteoglycan link protein 3(HAPLN3) Homo sapiens \| \| \| \| \|  \|  \|  \| \| HBE1 hemoglobin subunit epsilon 1(HBE1) Homo sapiens \| \| \|  \|  \|  \|  \|  \| \| HDAC4 histone deacetylase 4(HDAC4) Homo sapiens \| \| \|  \|  \|  \|  \|  \| \| HECW2 HECT, C2 and WW domain containing E3 ubiquitin protein ligase 2(HECW2) Homo sapiens \| \| \| \| \| \| \|  \| \| HERC6 HECT and RLD domain containing E3 ubiquitin protein ligase family member 6(HERC6) Homo sapiens \| \| \| \| \| \| \| \| \| HES1 hes family bHLH transcription factor 1(HES1) Homo sapiens \| \| \| \|  \|  \|  \|  \| \| HES4 hes family bHLH transcription factor 4(HES4) Homo sapiens \| \| \| \|  \|  \|  \|  \| \| HEY1 hes related family bHLH transcription factor with YRPW motif 1(HEY1) Homo sapiens \| \| \| \| \| \|  \|  \| \| HHEX hematopoietically expressed homeobox(HHEX) Homo sapiens \| \| \| \|  \|  \|  \|  \| \| HHIPL2 HHIP like 2(HHIPL2) Homo sapiens \| \|  \|  \|  \|  \|  \|  \| \| HILS1 histone linker H1 domain, spermatid-specific 1 (pseudogene)(HILS1) Homo sapiens \| \| \| \| \| \|  \|  \| \| HIVEP3 human immunodeficiency virus type I enhancer binding protein 3(HIVEP3) Homo sapiens \| \| \| \| \| \|  \|  \| \| HLA-DMA major histocompatibility complex, class II, DM alpha(HLA-DMA) Homo sapiens \| \| \| \| \| \|  \|  \| \| HLA-DMB major histocompatibility complex, class II, DM beta(HLA-DMB) Homo sapiens \| \| \| \| \| \|  \|  \| \| HLA-DOA major histocompatibility complex, class II, DO alpha(HLA-DOA) Homo sapiens \| \| \| \| \| \|  \|  \| \| HLA-DPA1 major histocompatibility complex, class II, DP alpha 1(HLA-DPA1) Homo sapiens \| \| \| \| \| \| \|  \| \| HLA-DQA1 major histocompatibility complex, class II, DQ alpha 1(HLA-DQA1) Homo sapiens \| \| \| \| \| \| \|  \| \| HLA-DQA2 major histocompatibility complex, class II, DQ alpha 2(HLA-DQA2) Homo sapiens \| \| \| \| \| \| \|  \| \| HLA-DQB1 major histocompatibility complex, class II, DQ beta 1(HLA-DQB1) Homo sapiens \| \| \| \| \| \|  \|  \| \| HLA-DQB2 major histocompatibility complex, class II, DQ beta 2(HLA-DQB2) Homo sapiens \| \| \| \| \| \|  \|  \| \| HLA-DRA major histocompatibility complex, class II, DR alpha(HLA-DRA) Homo sapiens \| \| \| \| \| \|  \|  \| \| HLA-DRB1 major histocompatibility complex, class II, DR beta 1(HLA-DRB1) Homo sapiens \| \| \| \| \| \|  \|  \| \| HLA-DRB5 major histocompatibility complex, class II, DR beta 5(HLA-DRB5) Homo sapiens \| \| \| \| \| \|  \|  \| \| HLX H2.0 like homeobox(HLX) Homo sapiens \| \|  \|  \|  \|  \|  \|  \| \| HMCN1 hemicentin 1(HMCN1) Homo sapiens \| \|  \|  \|  \|  \|  \|  \| \| HMGB1 high mobility group box 1(HMGB1) Homo sapiens \| \| \|  \|  \|  \|  \|  \| \| HOXB2 homeobox B2(HOXB2) Homo sapiens \| \|  \|  \|  \|  \|  \|  \| \| HOXD10 homeobox D10(HOXD10) Homo sapiens \| \|  \|  \|  \|  \|  \|  \| \| HPSE heparanase(HPSE) Homo sapiens \| \|  \|  \|  \|  \|  \|  \| \| HRASLS HRAS like suppressor(HRASLS) Homo sapiens \| \| \|  \|  \|  \|  \|  \| \| HSPB1 heat shock protein family B (small) member 1(HSPB1) Homo sapiens \| \| \| \| \|  \|  \|  \| \| HSPB3 heat shock protein family B (small) member 3(HSPB3) Homo sapiens \| \| \| \| \|  \|  \|  \| \| IFI30 IFI30, lysosomal thiol reductase(IFI30) Homo sapiens \| \| \|  \|  \|  \|  \|  \| \| IFI44 interferon induced protein 44(IFI44) Homo sapiens \| \| \|  \|  \|  \|  \|  \| \| IFIH1 interferon induced with helicase C domain 1(IFIH1) Homo sapiens \| \| \| \|  \|  \|  \|  \| \| IFIT1 interferon induced protein with tetratricopeptide repeats 1(IFIT1) Homo sapiens \| \| \| \| \| \|  \|  \| \| IFIT2 interferon induced protein with tetratricopeptide repeats 2(IFIT2) Homo sapiens \| \| \| \| \| \|  \|  \| \| IFIT3 interferon induced protein with tetratricopeptide repeats 3(IFIT3) Homo sapiens \| \| \| \| \| \|  \|  \| \| IFITM2 interferon induced transmembrane protein 2(IFITM2) Homo sapiens \| \| \| \| \|  \|  \|  \| \| IFNB1 interferon beta 1(IFNB1) Homo sapiens \| \|  \|  \|  \|  \|  \|  \| \| IFT88 intraflagellar transport 88(IFT88) Homo sapiens \| \| \|  \|  \|  \|  \|  \| \| IGF2BP2 insulin like growth factor 2 mRNA binding protein 2(IGF2BP2) Homo sapiens \| \| \| \| \| \|  \|  \| \| IGF2BP3 insulin like growth factor 2 mRNA binding protein 3(IGF2BP3) Homo sapiens \| \| \| \| \| \|  \|  \| \| IGFBP6 insulin like growth factor binding protein 6(IGFBP6) Homo sapiens \| \| \| \| \|  \|  \|  \| \| IGFN1 immunoglobulin-like and fibronectin type III domain containing 1(IGFN1) Homo sapiens \| \| \| \| \| \|  \|  \| \| IL15 interleukin 15(IL15) Homo sapiens \|  \|  \|  \|  \|  \|  \|  \| \| IL15RA interleukin 15 receptor subunit alpha(IL15RA) Homo sapiens \| \| \| \|  \|  \|  \|  \| \| IL16 interleukin 16(IL16) Homo sapiens \|  \|  \|  \|  \|  \|  \|  \| \| IL18 interleukin 18(IL18) Homo sapiens \|  \|  \|  \|  \|  \|  \|  \| \| IL2RG interleukin 2 receptor subunit gamma(IL2RG) Homo sapiens \| \| \| \|  \|  \|  \|  \| \| IL29 interleukin 29(IL29) Homo sapiens \|  \|  \|  \|  \|  \|  \|  \| \| IL31RA interleukin 31 receptor A(IL31RA) Homo sapiens \| \| \|  \|  \|  \|  \|  \| \| IL32 interleukin 32(IL32) Homo sapiens \|  \|  \|  \|  \|  \|  \|  \| \| IL6 interleukin 6(IL6) Homo sapiens \|  \|  \|  \|  \|  \|  \|  \| \| IL7R interleukin 7 receptor(IL7R) Homo sapiens \| \|  \|  \|  \|  \|  \|  \| \| ILDR2 immunoglobulin like domain containing receptor 2(ILDR2) Homo sapiens \| \| \| \| \|  \|  \|  \| \| INHBE inhibin beta E subunit(INHBE) Homo sapiens \| \| \|  \|  \|  \|  \|  \| \| INPP5F inositol polyphosphate-5-phosphatase F(INPP5F) Homo sapiens \| \| \| \|  \|  \|  \|  \| \| IRF2BPL interferon regulatory factor 2 binding protein like(IRF2BPL) Homo sapiens \| \| \| \| \|  \|  \|  \| \| IRS2 insulin receptor substrate 2(IRS2) Homo sapiens \| \| \|  \|  \|  \|  \|  \| \| IRX3 iroquois homeobox 3(IRX3) Homo sapiens \| \|  \|  \|  \|  \|  \|  \| \| IRX5 iroquois homeobox 5(IRX5) Homo sapiens \| \|  \|  \|  \|  \|  \|  \| \| IRX6 iroquois homeobox 6(IRX6) Homo sapiens \| \|  \|  \|  \|  \|  \|  \| \| ISG15 ISG15 ubiquitin-like modifier(ISG15) Homo sapiens \| \| \|  \|  \|  \|  \|  \| \| ISLR immunoglobulin superfamily containing leucine rich repeat(ISLR) Homo sapiens \| \| \| \| \|  \|  \|  \| \| ISYNA1 inositol-3-phosphate synthase 1(ISYNA1) Homo sapiens \| \| \|  \|  \|  \|  \|  \| \| ITGA3 integrin subunit alpha 3(ITGA3) Homo sapiens \| \|  \|  \|  \|  \|  \|  \| \| ITGA9 integrin subunit alpha 9(ITGA9) Homo sapiens \| \|  \|  \|  \|  \|  \|  \| \| ITGB2 integrin subunit beta 2(ITGB2) Homo sapiens \| \|  \|  \|  \|  \|  \|  \| \| ITGB8 integrin subunit beta 8(ITGB8) Homo sapiens \| \|  \|  \|  \|  \|  \|  \| \| ITIH5 inter-alpha-trypsin inhibitor heavy chain family member 5(ITIH5) Homo sapiens \| \| \| \| \| \|  \|  \| \| ITM2A integral membrane protein 2A(ITM2A) Homo sapiens \| \| \|  \|  \|  \|  \|  \| \| JAG1 jagged 1(JAG1) Homo sapiens \|  \|  \|  \|  \|  \|  \|  \| \| JDP2 Jun dimerization protein 2(JDP2) Homo sapiens \| \| \|  \|  \|  \|  \|  \| \| JPH2 junctophilin 2(JPH2) Homo sapiens \| \|  \|  \|  \|  \|  \|  \| \| JUND JunD proto-oncogene, AP-1 transcription factor subunit(JUND) Homo sapiens \| \| \| \| \| \|  \|  \| \| KCNC3 potassium voltage-gated channel subfamily C member 3(KCNC3) Homo sapiens \| \| \| \| \| \|  \|  \| \| KCNJ8 potassium voltage-gated channel subfamily J member 8(KCNJ8) Homo sapiens \| \| \| \| \| \|  \|  \| \| KCNK1 potassium two pore domain channel subfamily K member 1(KCNK1) Homo sapiens \| \| \| \| \| \|  \|  \| \| KCNK6 potassium two pore domain channel subfamily K member 6(KCNK6) Homo sapiens \| \| \| \| \| \|  \|  \| \| KDR kinase insert domain receptor(KDR) Homo sapiens \| \| \|  \|  \|  \|  \|  \| \| KHDRBS3 KH RNA binding domain containing, signal transduction associated 3(KHDRBS3) Homo sapiens \| \| \| \| \| \| \|  \| \| KIAA1161 KIAA1161(KIAA1161) Homo sapiens \| \| \|  \|  \|  \|  \|  \| \| KIAA1211 KIAA1211(KIAA1211) Homo sapiens \| \| \|  \|  \|  \|  \|  \| \| KIAA1522 KIAA1522(KIAA1522) Homo sapiens \| \| \|  \|  \|  \|  \|  \| \| KIFC2 kinesin family member C2(KIFC2) Homo sapiens \| \| \|  \|  \|  \|  \|  \| \| KRT34 keratin 34(KRT34) Homo sapiens \| \|  \|  \|  \|  \|  \|  \| \| KRT75 keratin 75(KRT75) Homo sapiens \| \|  \|  \|  \|  \|  \|  \| \| KRT8 keratin 8(KRT8) Homo sapiens \|  \|  \|  \|  \|  \|  \|  \| \| KRT81 keratin 81(KRT81) Homo sapiens \| \|  \|  \|  \|  \|  \|  \| \| L1CAM L1 cell adhesion molecule(L1CAM) Homo sapiens \| \| \|  \|  \|  \|  \|  \| \| LAMA5 laminin subunit alpha 5(LAMA5) Homo sapiens \| \| \|  \|  \|  \|  \|  \| \| LDLR low density lipoprotein receptor(LDLR) Homo sapiens \| \| \|  \|  \|  \|  \|  \| \| LEF1 lymphoid enhancer binding factor 1(LEF1) Homo sapiens \| \| \| \|  \|  \|  \|  \| \| LHPP phospholysine phosphohistidine inorganic pyrophosphate phosphatase(LHPP) Homo sapiens \| \| \| \| \| \| \|  \| \| LIMS2 LIM zinc finger domain containing 2(LIMS2) Homo sapiens \| \| \| \|  \|  \|  \|  \| \| LINC00189 long intergenic non-protein coding RNA 189(LINC00189) Homo sapiens \| \| \| \| \|  \|  \|  \| \| LINC00326 long intergenic non-protein coding RNA 326(LINC00326) Homo sapiens \| \| \| \| \|  \|  \|  \| \| LINC00346 long intergenic non-protein coding RNA 346(LINC00346) Homo sapiens \| \| \| \| \|  \|  \|  \| \| LINC00473 long intergenic non-protein coding RNA 473(LINC00473) Homo sapiens \| \| \| \| \|  \|  \|  \| \| LINC00525 long intergenic non-protein coding RNA 525(LINC00525) Homo sapiens \| \| \| \| \|  \|  \|  \| \| LINC00589 long intergenic non-protein coding RNA 589(LINC00589) Homo sapiens \| \| \| \| \|  \|  \|  \| \| LMO4 LIM domain only 4(LMO4) Homo sapiens \| \|  \|  \|  \|  \|  \|  \| \| LOC100133985 uncharacterized LOC100133985(LOC100133985) Homo sapiens \| \| \| \| \|  \|  \|  \| \| LOC100268168 uncharacterized LOC100268168(LOC100268168) Homo sapiens \| \| \| \| \|  \|  \|  \| \| LOC100506474 uncharacterized LOC100506474(LOC100506474) Homo sapiens \| \| \| \| \|  \|  \|  \| \| LOC152225 uncharacterized LOC152225(LOC152225) Homo sapiens \| \| \| \|  \|  \|  \|  \| \| LOC389033 placenta specific 9 pseudogene(LOC389033) Homo sapiens \| \| \| \| \|  \|  \|  \| \| LOC554223 histocompatibility antigen-related(LOC554223) Homo sapiens \| \| \| \| \|  \|  \|  \| \| LOC645166 lymphocyte-specific protein 1 pseudogene(LOC645166) Homo sapiens \| \| \| \| \| \|  \|  \| \| LOC646268 hCG1654703(LOC646268) Homo sapiens \| \| \|  \|  \|  \|  \|  \| \| LOC654342 lymphocyte-specific protein 1 pseudogene(LOC654342) Homo sapiens \| \| \| \| \| \|  \|  \| \| LOC728392 uncharacterized LOC728392(LOC728392) Homo sapiens \| \| \| \|  \|  \|  \|  \| \| LOC730102 quinone oxidoreductase-like protein 2 pseudogene(LOC730102) Homo sapiens \| \| \| \| \| \|  \|  \| \| LONRF3 LON peptidase N-terminal domain and ring finger 3(LONRF3) Homo sapiens \| \| \| \| \| \|  \|  \| \| LOXL1 lysyl oxidase like 1(LOXL1) Homo sapiens \| \|  \|  \|  \|  \|  \|  \| \| LPAR5 lysophosphatidic acid receptor 5(LPAR5) Homo sapiens \| \| \|  \|  \|  \|  \|  \| \| LPAR6 lysophosphatidic acid receptor 6(LPAR6) Homo sapiens \| \| \|  \|  \|  \|  \|  \| \| LPHN2 latrophilin-2 Homo Sapiens \|  \|  \|  \|  \|  \|  \|  \| \| LRAT lecithin retinol acyltransferase (phosphatidylcholine--retinol O-acyltransferase)(LRAT) Homo sapiens \| \| \| \| \| \| \|  \| \| LRP1 LDL receptor related protein 1(LRP1) Homo sapiens \| \| \|  \|  \|  \|  \|  \| \| LRRC17 leucine rich repeat containing 17(LRRC17) Homo sapiens \| \| \| \|  \|  \|  \|  \| \| LRRC7 leucine rich repeat containing 7(LRRC7) Homo sapiens \| \| \|  \|  \|  \|  \|  \| \| LRRK2 leucine rich repeat kinase 2(LRRK2) Homo sapiens \| \| \|  \|  \|  \|  \|  \| \| LUM lumican(LUM) Homo sapiens \|  \|  \|  \|  \|  \|  \|  \| \| LURAP1L leucine rich adaptor protein 1 like(LURAP1L) Homo sapiens \| \| \| \|  \|  \|  \|  \| \| MAFB MAF bZIP transcription factor B(MAFB) Homo sapiens \| \| \|  \|  \|  \|  \|  \| \| MAGEA10 MAGE family member A10(MAGEA10) Homo sapiens \| \| \|  \|  \|  \|  \|  \| \| MAML3 mastermind like transcriptional coactivator 3(MAML3) Homo sapiens \| \| \| \| \|  \|  \|  \| \| MANSC1 MANSC domain containing 1(MANSC1) Homo sapiens \| \| \| \|  \|  \|  \|  \| \| MAP3K1 mitogen-activated protein kinase kinase kinase 1(MAP3K1) Homo sapiens \| \| \| \| \| \|  \|  \| \| MAPT microtubule associated protein tau(MAPT) Homo sapiens \| \| \| \|  \|  \|  \|  \| \| MEGF10 multiple EGF like domains 10(MEGF10) Homo sapiens \| \| \| \|  \|  \|  \|  \| \| MEGF6 multiple EGF like domains 6(MEGF6) Homo sapiens \| \| \| \|  \|  \|  \|  \| \| METRNL meteorin like, glial cell differentiation regulator(METRNL) Homo sapiens \| \| \| \| \|  \|  \|  \| \| MGC16025 uncharacterized LOC85009(MGC16025) Homo sapiens \| \| \| \|  \|  \|  \|  \| \| MGC72080 MGC72080 pseudogene(MGC72080) Homo sapiens \| \| \| \|  \|  \|  \|  \| \| MGP matrix Gla protein(MGP) Homo sapiens \| \|  \|  \|  \|  \|  \|  \| \| MIA melanoma inhibitory activity(MIA) Homo sapiens \| \| \|  \|  \|  \|  \|  \| \| MIA-RAB4B MIA-RAB4B readthrough (NMD candidate)(MIA-RAB4B) Homo sapiens \| \| \| \| \| \|  \|  \| \| MICAL2 microtubule associated monooxygenase, calponin and LIM domain containing 2(MICAL2) Homo sapiens \| \| \| \| \| \| \| \| \| MME membrane metalloendopeptidase(MME) Homo sapiens \| \| \| \|  \|  \|  \|  \| \| MMP1 matrix metallopeptidase 1(MMP1) Homo sapiens \| \| \|  \|  \|  \|  \|  \| \| MMP16 matrix metallopeptidase 16(MMP16) Homo sapiens \| \| \|  \|  \|  \|  \|  \| \| MMP17 matrix metallopeptidase 17(MMP17) Homo sapiens \| \| \|  \|  \|  \|  \|  \| \| MMP2 matrix metallopeptidase 2(MMP2) Homo sapiens \| \| \|  \|  \|  \|  \|  \| \| MMP24 matrix metallopeptidase 24(MMP24) Homo sapiens \| \| \|  \|  \|  \|  \|  \| \| MMP8 matrix metallopeptidase 8(MMP8) Homo sapiens \| \| \|  \|  \|  \|  \|  \| \| MMP9 matrix metallopeptidase 9(MMP9) Homo sapiens \| \| \|  \|  \|  \|  \|  \| \| MOK MOK protein kinase(MOK) Homo sapiens \| \|  \|  \|  \|  \|  \|  \| \| MPP7 membrane palmitoylated protein 7(MPP7) Homo sapiens \| \| \| \|  \|  \|  \|  \| \| MPZ myelin protein zero(MPZ) Homo sapiens \| \|  \|  \|  \|  \|  \|  \| \| MRAS muscle RAS oncogene homolog(MRAS) Homo sapiens \| \| \| \|  \|  \|  \|  \| \| MRGPRX4 MAS related GPR family member X4(MRGPRX4) Homo sapiens \| \| \| \| \|  \|  \|  \| \| MSR1 macrophage scavenger receptor 1(MSR1) Homo sapiens \| \| \| \|  \|  \|  \|  \| \| MT1F metallothionein 1F(MT1F) Homo sapiens \| \|  \|  \|  \|  \|  \|  \| \| MTL5 metallothionein-Like 5, Testis-Specific (Tesmin) Homo Sapiens \| \| \| \|  \|  \|  \|  \| \| MUC1 mucin 1, cell surface associated(MUC1) Homo sapiens \| \| \|  \|  \|  \|  \|  \| \| MUC13 mucin 13, cell surface associated(MUC13) Homo sapiens \| \| \| \|  \|  \|  \|  \| \| MUC5B mucin 5B, oligomeric mucus/gel-forming(MUC5B) Homo sapiens \| \| \| \|  \|  \|  \|  \| \| MX1 MX dynamin like GTPase 1(MX1) Homo sapiens \| \| \|  \|  \|  \|  \|  \| \| MXRA7 matrix remodeling associated 7(MXRA7) Homo sapiens \| \| \| \|  \|  \|  \|  \| \| MYL9 myosin light chain 9(MYL9) Homo sapiens \| \|  \|  \|  \|  \|  \|  \| \| MYLK myosin light chain kinase(MYLK) Homo sapiens \| \| \|  \|  \|  \|  \|  \| \| MYO7B myosin VIIB(MYO7B) Homo sapiens \| \|  \|  \|  \|  \|  \|  \| \| MYOCD myocardin(MYOCD) Homo sapiens \| \|  \|  \|  \|  \|  \|  \| \| MYZAP myocardial zonula adherens protein(MYZAP) Homo sapiens \| \| \| \|  \|  \|  \|  \| \| NAB2 NGFI-A binding protein 2(NAB2) Homo sapiens \| \| \|  \|  \|  \|  \|  \| \| NACC2 NACC family member 2(NACC2) Homo sapiens \| \| \|  \|  \|  \|  \|  \| \| NAP1L2 nucleosome assembly protein 1 like 2(NAP1L2) Homo sapiens \| \| \| \|  \|  \|  \|  \| \| NAT8L N-acetyltransferase 8 like(NAT8L) Homo sapiens \| \| \|  \|  \|  \|  \|  \| \| NCAM1 neural cell adhesion molecule 1(NCAM1) Homo sapiens \| \| \| \|  \|  \|  \|  \| \| NCF2 neutrophil cytosolic factor 2(NCF2) Homo sapiens \| \| \|  \|  \|  \|  \|  \| \| NEK3 NIMA related kinase 3(NEK3) Homo sapiens \| \| \|  \|  \|  \|  \|  \| \| NES nestin(NES) Homo sapiens \|  \|  \|  \|  \|  \|  \|  \| \| NETO1 neuropilin and tolloid like 1(NETO1) Homo sapiens \| \| \|  \|  \|  \|  \|  \| \| NEXN nexilin F-actin binding protein(NEXN) Homo sapiens \| \| \|  \|  \|  \|  \|  \| \| NFASC neurofascin(NFASC) Homo sapiens \| \|  \|  \|  \|  \|  \|  \| \| NFATC1 nuclear factor of activated T-cells 1(NFATC1) Homo sapiens \| \| \| \|  \|  \|  \|  \| \| NFATC2 nuclear factor of activated T-cells 2(NFATC2) Homo sapiens \| \| \| \|  \|  \|  \|  \| \| NGF nerve growth factor(NGF) Homo sapiens \| \|  \|  \|  \|  \|  \|  \| \| NHSL1 NHS like 1(NHSL1) Homo sapiens \| \|  \|  \|  \|  \|  \|  \| \| NID2 nidogen 2(NID2) Homo sapiens \|  \|  \|  \|  \|  \|  \|  \| \| NINJ1 ninjurin 1(NINJ1) Homo sapiens \| \|  \|  \|  \|  \|  \|  \| \| NINL ninein like(NINL) Homo sapiens \|  \|  \|  \|  \|  \|  \|  \| \| NMNAT2 nicotinamide nucleotide adenylyltransferase 2(NMNAT2) Homo sapiens \| \| \| \| \|  \|  \|  \| \| NPAS2 neuronal PAS domain protein 2(NPAS2) Homo sapiens \| \| \| \|  \|  \|  \|  \| \| NPB neuropeptide B(NPB) Homo sapiens \| \|  \|  \|  \|  \|  \|  \| \| NPPC natriuretic peptide C(NPPC) Homo sapiens \| \|  \|  \|  \|  \|  \|  \| \| NPTX2 neuronal pentraxin 2(NPTX2) Homo sapiens \| \| \|  \|  \|  \|  \|  \| \| NPTXR neuronal pentraxin receptor(NPTXR) Homo sapiens \| \| \|  \|  \|  \|  \|  \| \| NR3C2 nuclear receptor subfamily 3 group C member 2(NR3C2) Homo sapiens \| \| \| \| \|  \|  \|  \| \| NR4A1 nuclear receptor subfamily 4 group A member 1(NR4A1) Homo sapiens \| \| \| \| \|  \|  \|  \| \| NR4A2 nuclear receptor subfamily 4 group A member 2(NR4A2) Homo sapiens \| \| \| \| \|  \|  \|  \| \| NRCAM neuronal cell adhesion molecule(NRCAM) Homo sapiens \| \| \| \|  \|  \|  \|  \| \| NREP neuronal regeneration related protein(NREP) Homo sapiens \| \| \| \|  \|  \|  \|  \| \| NRG1 neuregulin 1(NRG1) Homo sapiens \| \|  \|  \|  \|  \|  \|  \| \| NRIP3 nuclear receptor interacting protein 3(NRIP3) Homo sapiens \| \| \| \|  \|  \|  \|  \| \| NTF3 neurotrophin 3(NTF3) Homo sapiens \| \|  \|  \|  \|  \|  \|  \| \| NTF4 neurotrophin 4(NTF4) Homo sapiens \| \|  \|  \|  \|  \|  \|  \| \| NTM neurotrimin(NTM) Homo sapiens \| \|  \|  \|  \|  \|  \|  \| \| NTN4 netrin 4(NTN4) Homo sapiens \|  \|  \|  \|  \|  \|  \|  \| \| NTNG1 netrin G1(NTNG1) Homo sapiens \| \|  \|  \|  \|  \|  \|  \| \| NUAK1 NUAK family kinase 1(NUAK1) Homo sapiens \| \| \|  \|  \|  \|  \|  \| \| NUPR1 nuclear protein 1, transcriptional regulator(NUPR1) Homo sapiens \| \| \| \| \|  \|  \|  \| \| NXNL2 nucleoredoxin-like 2(NXNL2) Homo sapiens \| \| \|  \|  \|  \|  \|  \| \| OASL 2'-5'-oligoadenylate synthetase like(OASL) Homo sapiens \| \| \| \|  \|  \|  \|  \| \| ODZ2 Protein Odd Oz/Ten-M Homolog 2 or Teneurin Transmembrane Protein 2 (TENM2) Homo Sapiens \| \| \| \| \| \| \| \| \| ODZ3 Protein Odd Oz/Ten-M Homolog 3 or Teneurin Transmembrane Protein 3 (TENM3) Homo Sapiens \| \| \| \| \| \| \| \| \| OLR1 oxidized low density lipoprotein receptor 1(OLR1) Homo sapiens \| \| \| \|  \|  \|  \|  \| \| OPRD1 opioid receptor delta 1(OPRD1) Homo sapiens \| \| \|  \|  \|  \|  \|  \| \| OPTN optineurin(OPTN) Homo sapiens \| \|  \|  \|  \|  \|  \|  \| \| OR2T8 olfactory receptor family 2 subfamily T member 8(OR2T8) Homo sapiens \| \| \| \| \|  \|  \|  \| \| OR51B4 olfactory receptor family 51 subfamily B member 4(OR51B4) Homo sapiens \| \| \| \| \| \|  \|  \| \| OR51B5 olfactory receptor family 51 subfamily B member 5(OR51B5) Homo sapiens \| \| \| \| \| \|  \|  \| \| OR51B6 olfactory receptor family 51 subfamily B member 6(OR51B6) Homo sapiens \| \| \| \| \| \|  \|  \| \| OR51I2 olfactory receptor family 51 subfamily I member 2(OR51I2) Homo sapiens \| \| \| \| \|  \|  \|  \| \| OR6F1 olfactory receptor family 6 subfamily F member 1(OR6F1) Homo sapiens \| \| \| \| \|  \|  \|  \| \| P2RY1 purinergic receptor P2Y1(P2RY1) Homo sapiens \| \| \|  \|  \|  \|  \|  \| \| PAPPA2 pappalysin 2(PAPPA2) Homo sapiens \| \|  \|  \|  \|  \|  \|  \| \| PARP10 poly(ADP-ribose) polymerase family member 10(PARP10) Homo sapiens \| \| \| \| \|  \|  \|  \| \| PBX1 PBX homeobox 1(PBX1) Homo sapiens \| \|  \|  \|  \|  \|  \|  \| \| PBXIP1 PBX homeobox interacting protein 1(PBXIP1) Homo sapiens \| \| \| \|  \|  \|  \|  \| \| PCBP3 poly(rC) binding protein 3(PCBP3) Homo sapiens \| \| \|  \|  \|  \|  \|  \| \| PCDH1 protocadherin 1(PCDH1) Homo sapiens \| \|  \|  \|  \|  \|  \|  \| \| PCDHB8 protocadherin beta 8(PCDHB8) Homo sapiens \| \| \|  \|  \|  \|  \|  \| \| PDCD1LG2 programmed cell death 1 ligand 2(PDCD1LG2) Homo sapiens \| \| \| \| \|  \|  \|  \| \| PDE5A phosphodiesterase 5A(PDE5A) Homo sapiens \| \| \|  \|  \|  \|  \|  \| \| PDGFC platelet derived growth factor C(PDGFC) Homo sapiens \| \| \|  \|  \|  \|  \|  \| \| PDGFD platelet derived growth factor D(PDGFD) Homo sapiens \| \| \|  \|  \|  \|  \|  \| \| PDLIM1 PDZ and LIM domain 1(PDLIM1) Homo sapiens \| \| \|  \|  \|  \|  \|  \| \| PFKFB3 6-phosphofructo-2-kinase/fructose-2,6-biphosphatase 3(PFKFB3) Homo sapiens \| \| \| \| \| \|  \|  \| \| PGBD5 piggyBac transposable element derived 5(PGBD5) Homo sapiens \| \| \| \|  \|  \|  \|  \| \| PGF placental growth factor(PGF) Homo sapiens \| \|  \|  \|  \|  \|  \|  \| \| PHC2 polyhomeotic homolog 2(PHC2) Homo sapiens \| \| \|  \|  \|  \|  \|  \| \| PHLDA1 pleckstrin homology like domain family A member 1(PHLDA1) Homo sapiens \| \| \| \| \| \|  \|  \| \| PHLDA2 pleckstrin homology like domain family A member 2(PHLDA2) Homo sapiens \| \| \| \| \| \|  \|  \| \| PHOSPHO2-KLHL23 PHOSPHO2-KLHL23 readthrough(PHOSPHO2-KLHL23) Homo sapiens \| \| \| \| \| \|  \|  \| \| PI15 peptidase inhibitor 15(PI15) Homo sapiens \| \|  \|  \|  \|  \|  \|  \| \| PI3 peptidase inhibitor 3(PI3) Homo sapiens \| \|  \|  \|  \|  \|  \|  \| \| PINK1 PTEN induced putative kinase 1(PINK1) Homo sapiens \| \| \|  \|  \|  \|  \|  \| \| PITPNM3 PITPNM family member 3(PITPNM3) Homo sapiens \| \| \|  \|  \|  \|  \|  \| \| PLA2G4C phospholipase A2 group IVC(PLA2G4C) Homo sapiens \| \| \| \|  \|  \|  \|  \| \| PLAC8 placenta specific 8(PLAC8) Homo sapiens \| \| \|  \|  \|  \|  \|  \| \| PLEKHA2 pleckstrin homology domain containing A2(PLEKHA2) Homo sapiens \| \| \| \| \|  \|  \|  \| \| PLEKHB1 pleckstrin homology domain containing B1(PLEKHB1) Homo sapiens \| \| \| \| \|  \|  \|  \| \| PLEKHG1 pleckstrin homology and RhoGEF domain containing G1(PLEKHG1) Homo sapiens \| \| \| \| \| \|  \|  \| \| PLLP plasmolipin(PLLP) Homo sapiens \| \|  \|  \|  \|  \|  \|  \| \| PLOD2 procollagen-lysine,2-oxoglutarate 5-dioxygenase 2(PLOD2) Homo sapiens \| \| \| \| \| \|  \|  \| \| PLP1 proteolipid protein 1(PLP1) Homo sapiens \| \|  \|  \|  \|  \|  \|  \| \| PLXDC2 plexin domain containing 2(PLXDC2) Homo sapiens \| \| \|  \|  \|  \|  \|  \| \| PMP22 peripheral myelin protein 22(PMP22) Homo sapiens \| \| \|  \|  \|  \|  \|  \| \| POSTN periostin(POSTN) Homo sapiens \| \|  \|  \|  \|  \|  \|  \| \| POU3F2 POU class 3 homeobox 2(POU3F2) Homo sapiens \| \| \|  \|  \|  \|  \|  \| \| PPAPDC1A phosphatidic Acid Phosphatase Type 2 Domain Containing 1A Homo Sapiens \| \| \| \| \| \|  \|  \| \| PPM1H protein phosphatase, Mg2+/Mn2+ dependent 1H(PPM1H) Homo sapiens \| \| \| \| \|  \|  \|  \| \| PPYR1 pancreatic Polypeptide Receptor 1 Homo Sapiens \| \| \|  \|  \|  \|  \|  \| \| PRKCD protein kinase C delta(PRKCD) Homo sapiens \| \| \|  \|  \|  \|  \|  \| \| PROCR protein C receptor(PROCR) Homo sapiens \| \| \|  \|  \|  \|  \|  \| \| ProSAPiP1 ProSAP-Interacting Protein 1 Homo Sapiens \| \| \| \| \| \| \| \| \| PRR4 proline rich 4 (lacrimal)(PRR4) Homo sapiens \| \|  \|  \|  \|  \|  \|  \| \| PRR5L proline rich 5 like(PRR5L) Homo sapiens \| \|  \|  \|  \|  \|  \|  \| \| PRRX1 paired related homeobox 1(PRRX1) Homo sapiens \| \| \|  \|  \|  \|  \|  \| \| PRUNE2 prune homolog 2(PRUNE2) Homo sapiens \| \|  \|  \|  \|  \|  \|  \| \| PSG4 pregnancy specific beta-1-glycoprotein 4(PSG4) Homo sapiens \| \| \| \|  \|  \|  \|  \| \| PSORS1C1 psoriasis susceptibility 1 candidate 1(PSORS1C1) Homo sapiens \| \| \| \| \|  \|  \|  \| \| PTCH1 patched 1(PTCH1) Homo sapiens \| \|  \|  \|  \|  \|  \|  \| \| PTGFR prostaglandin F receptor(PTGFR) Homo sapiens \| \| \|  \|  \|  \|  \|  \| \| PTPLA protein Tyrosine Phosphatase-Like (Proline Instead Of Catalytic Arginine), Member A Homo Sapiens \| \| \| \| \| \| \| \| \| PTPRB protein tyrosine phosphatase, receptor type B(PTPRB) Homo sapiens \| \| \| \| \|  \|  \|  \| \| PTPRN protein tyrosine phosphatase, receptor type N(PTPRN) Homo sapiens \| \| \| \| \|  \|  \|  \| \| PYGB phosphorylase, glycogen; brain(PYGB) Homo sapiens \| \| \|  \|  \|  \|  \|  \| \| QSOX1 quiescin sulfhydryl oxidase 1(QSOX1) Homo sapiens \| \| \|  \|  \|  \|  \|  \| \| RAB39A RAB39A, member RAS oncogene family(RAB39A) Homo sapiens \| \| \| \| \|  \|  \|  \| \| RAB3B RAB3B, member RAS oncogene family(RAB3B) Homo sapiens \| \| \| \| \|  \|  \|  \| \| RARRES3 retinoic acid receptor responder 3(RARRES3) Homo sapiens \| \| \| \|  \|  \|  \|  \| \| RASA2 RAS p21 protein activator 2(RASA2) Homo sapiens \| \| \|  \|  \|  \|  \|  \| \| RASSF2 Ras association domain family member 2(RASSF2) Homo sapiens \| \| \| \| \|  \|  \|  \| \| RASSF8 Ras association domain family member 8(RASSF8) Homo sapiens \| \| \| \| \|  \|  \|  \| \| RBM24 RNA binding motif protein 24(RBM24) Homo sapiens \| \| \|  \|  \|  \|  \|  \| \| RDM1 RAD52 motif containing 1(RDM1) Homo sapiens \| \| \|  \|  \|  \|  \|  \| \| REP15 RAB15 effector protein(REP15) Homo sapiens \| \| \|  \|  \|  \|  \|  \| \| RGMA repulsive guidance molecule family member a(RGMA) Homo sapiens \| \| \| \| \|  \|  \|  \| \| RGS20 regulator of G-protein signaling 20(RGS20) Homo sapiens \| \| \| \|  \|  \|  \|  \| \| RHOB ras homolog family member B(RHOB) Homo sapiens \| \| \| \|  \|  \|  \|  \| \| RHOJ ras homolog family member J(RHOJ) Homo sapiens \| \| \|  \|  \|  \|  \|  \| \| RIMS2 regulating synaptic membrane exocytosis 2(RIMS2) Homo sapiens \| \| \| \| \|  \|  \|  \| \| RIMS3 regulating synaptic membrane exocytosis 3(RIMS3) Homo sapiens \| \| \| \| \|  \|  \|  \| \| RNF125 ring finger protein 125(RNF125) Homo sapiens \| \| \|  \|  \|  \|  \|  \| \| RNF157 ring finger protein 157(RNF157) Homo sapiens \| \| \|  \|  \|  \|  \|  \| \| ROPN1 rhophilin associated tail protein 1(ROPN1) Homo sapiens \| \| \| \|  \|  \|  \|  \| \| ROS1 ROS proto-oncogene 1, receptor tyrosine kinase(ROS1) Homo sapiens \| \| \| \| \|  \|  \|  \| \| RPS6KA5 ribosomal protein S6 kinase A5(RPS6KA5) Homo sapiens \| \| \| \|  \|  \|  \|  \| \| RRAS related RAS viral (r-ras) oncogene homolog(RRAS) Homo sapiens \| \| \| \| \|  \|  \|  \| \| RSAD2 radical S-adenosyl methionine domain containing 2(RSAD2) Homo sapiens \| \| \| \| \| \|  \|  \| \| RTP3 receptor transporter protein 3(RTP3) Homo sapiens \| \| \|  \|  \|  \|  \|  \| \| RUNX1T1 RUNX1 translocation partner 1(RUNX1T1) Homo sapiens \| \| \| \|  \|  \|  \|  \| \| RUNX2 runt related transcription factor 2(RUNX2) Homo sapiens \| \| \| \|  \|  \|  \|  \| \| RXRG retinoid X receptor gamma(RXRG) Homo sapiens \| \| \|  \|  \|  \|  \|  \| \| S100A1 S100 calcium binding protein A1(S100A1) Homo sapiens \| \| \| \|  \|  \|  \|  \| \| S100B S100 calcium binding protein B(S100B) Homo sapiens \| \| \|  \|  \|  \|  \|  \| \| S1PR2 sphingosine-1-phosphate receptor 2(S1PR2) Homo sapiens \| \| \| \|  \|  \|  \|  \| \| SAMD11 sterile alpha motif domain containing 11(SAMD11) Homo sapiens \| \| \| \| \|  \|  \|  \| \| SAMD12 sterile alpha motif domain containing 12(SAMD12) Homo sapiens \| \| \| \| \|  \|  \|  \| \| SAMD9L sterile alpha motif domain containing 9 like(SAMD9L) Homo sapiens \| \| \| \| \|  \|  \|  \| \| SBSN suprabasin(SBSN) Homo sapiens \| \|  \|  \|  \|  \|  \|  \| \| SCG2 secretogranin II(SCG2) Homo sapiens \| \|  \|  \|  \|  \|  \|  \| \| SCG5 secretogranin V(SCG5) Homo sapiens \| \|  \|  \|  \|  \|  \|  \| \| SCHIP1 schwannomin interacting protein 1(SCHIP1) Homo sapiens \| \| \| \|  \|  \|  \|  \| \| SDC3 syndecan 3(SDC3) Homo sapiens \| \|  \|  \|  \|  \|  \|  \| \| SECTM1 secreted and transmembrane 1(SECTM1) Homo sapiens \| \| \| \|  \|  \|  \|  \| \| SEL1L3 SEL1L family member 3(SEL1L3) Homo sapiens \| \| \|  \|  \|  \|  \|  \| \| SELM selenoprotein M Homo Sapiens \|  \|  \|  \|  \|  \|  \|  \| \| SEMA4C semaphorin 4C(SEMA4C) Homo sapiens \| \|  \|  \|  \|  \|  \|  \| \| SEPT6 septin 6(SEPT6) Homo sapiens \|  \|  \|  \|  \|  \|  \|  \| \| SERINC2 serine incorporator 2(SERINC2) Homo sapiens \| \| \|  \|  \|  \|  \|  \| \| SERPINA1 serpin family A member 1(SERPINA1) Homo sapiens \| \| \| \|  \|  \|  \|  \| \| SERPINA3 serpin family A member 3(SERPINA3) Homo sapiens \| \| \| \|  \|  \|  \|  \| \| SERPINA5 serpin family A member 5(SERPINA5) Homo sapiens \| \| \| \|  \|  \|  \|  \| \| SERPINB7 serpin family B member 7(SERPINB7) Homo sapiens \| \| \| \|  \|  \|  \|  \| \| SERPINE1 serpin family E member 1(SERPINE1) Homo sapiens \| \| \| \|  \|  \|  \|  \| \| SERPINE2 serpin family E member 2(SERPINE2) Homo sapiens \| \| \| \|  \|  \|  \|  \| \| SERPINF1 serpin family F member 1(SERPINF1) Homo sapiens \| \| \| \|  \|  \|  \|  \| \| SERTAD4 SERTA domain containing 4(SERTAD4) Homo sapiens \| \| \| \|  \|  \|  \|  \| \| SFN stratifin(SFN) Homo sapiens \|  \|  \|  \|  \|  \|  \|  \| \| SFRP1 secreted frizzled related protein 1(SFRP1) Homo sapiens \| \| \| \|  \|  \|  \|  \| \| SFTA1P surfactant associated 1, pseudogene(SFTA1P) Homo sapiens \| \| \| \|  \|  \|  \|  \| \| SH2B3 SH2B adaptor protein 3(SH2B3) Homo sapiens \| \| \|  \|  \|  \|  \|  \| \| SH3BP4 SH3 domain binding protein 4(SH3BP4) Homo sapiens \| \| \| \|  \|  \|  \|  \| \| SH3GL2 SH3 domain containing GRB2 like 2, endophilin A1(SH3GL2) Homo sapiens \| \| \| \| \| \|  \|  \| \| SH3RF2 SH3 domain containing ring finger 2(SH3RF2) Homo sapiens \| \| \| \|  \|  \|  \|  \| \| SH3TC2 SH3 domain and tetratricopeptide repeats 2(SH3TC2) Homo sapiens \| \| \| \| \|  \|  \|  \| \| SHC2 SHC adaptor protein 2(SHC2) Homo sapiens \| \| \|  \|  \|  \|  \|  \| \| SHC4 SHC adaptor protein 4(SHC4) Homo sapiens \| \| \|  \|  \|  \|  \|  \| \| SHROOM2 shroom family member 2(SHROOM2) Homo sapiens \| \| \|  \|  \|  \|  \|  \| \| SHROOM4 shroom family member 4(SHROOM4) Homo sapiens \| \| \|  \|  \|  \|  \|  \| \| SLAIN1 SLAIN motif family member 1(SLAIN1) Homo sapiens \| \| \|  \|  \|  \|  \|  \| \| SLAMF7 SLAM family member 7(SLAMF7) Homo sapiens \| \| \|  \|  \|  \|  \|  \| \| SLC12A8 solute carrier family 12 member 8(SLC12A8) Homo sapiens \| \| \| \|  \|  \|  \|  \| \| SLC16A2 solute carrier family 16 member 2(SLC16A2) Homo sapiens \| \| \| \|  \|  \|  \|  \| \| SLC16A9 solute carrier family 16 member 9(SLC16A9) Homo sapiens \| \| \| \|  \|  \|  \|  \| \| SLC20A1 solute carrier family 20 member 1(SLC20A1) Homo sapiens \| \| \| \|  \|  \|  \|  \| \| SLC26A2 solute carrier family 26 member 2(SLC26A2) Homo sapiens \| \| \| \|  \|  \|  \|  \| \| SLC2A3 solute carrier family 2 member 3(SLC2A3) Homo sapiens \| \| \| \|  \|  \|  \|  \| \| SLC35F1 solute carrier family 35 member F1(SLC35F1) Homo sapiens \| \| \| \|  \|  \|  \|  \| \| SLC38A4 solute carrier family 38 member 4(SLC38A4) Homo sapiens \| \| \| \|  \|  \|  \|  \| \| SLC4A11 solute carrier family 4 member 11(SLC4A11) Homo sapiens \| \| \| \|  \|  \|  \|  \| \| SLC4A4 solute carrier family 4 member 4(SLC4A4) Homo sapiens \| \| \| \|  \|  \|  \|  \| \| SLC8A1 solute carrier family 8 member A1(SLC8A1) Homo sapiens \| \| \| \|  \|  \|  \|  \| \| SLC9A9 solute carrier family 9 member A9(SLC9A9) Homo sapiens \| \| \| \|  \|  \|  \|  \| \| SLITRK6 SLIT and NTRK like family member 6(SLITRK6) Homo sapiens \| \| \| \|  \|  \|  \|  \| \| SLPI secretory leukocyte peptidase inhibitor(SLPI) Homo sapiens \| \| \| \|  \|  \|  \|  \| \| SMPDL3B sphingomyelin phosphodiesterase acid like 3B(SMPDL3B) Homo sapiens \| \| \| \| \|  \|  \|  \| \| SNED1 sushi, nidogen and EGF like domains 1(SNED1) Homo sapiens \| \| \| \|  \|  \|  \|  \| \| SNPH syntaphilin(SNPH) Homo sapiens \| \|  \|  \|  \|  \|  \|  \| \| SNRPB2 small nuclear ribonucleoprotein polypeptide B2(SNRPB2) Homo sapiens \| \| \| \| \|  \|  \|  \| \| SORBS2 sorbin and SH3 domain containing 2(SORBS2) Homo sapiens \| \| \| \|  \|  \|  \|  \| \| SORCS1 sortilin related VPS10 domain containing receptor 1(SORCS1) Homo sapiens \| \| \| \| \| \|  \|  \| \| SOX10 SRY-box 10(SOX10) Homo sapiens \| \|  \|  \|  \|  \|  \|  \| \| SOX11 SRY-box 11(SOX11) Homo sapiens \| \|  \|  \|  \|  \|  \|  \| \| SOX5 SRY-box 5(SOX5) Homo sapiens \|  \|  \|  \|  \|  \|  \|  \| \| SOX6 SRY-box 6(SOX6) Homo sapiens \|  \|  \|  \|  \|  \|  \|  \| \| SOX8 SRY-box 8(SOX8) Homo sapiens \|  \|  \|  \|  \|  \|  \|  \| \| SOX9 SRY-box 9(SOX9) Homo sapiens \|  \|  \|  \|  \|  \|  \|  \| \| SP140 SP140 nuclear body protein(SP140) Homo sapiens \| \| \|  \|  \|  \|  \|  \| \| SPAG4 sperm associated antigen 4(SPAG4) Homo sapiens \| \| \|  \|  \|  \|  \|  \| \| SPATA13 spermatogenesis associated 13(SPATA13) Homo sapiens \| \| \| \|  \|  \|  \|  \| \| SPATA4 spermatogenesis associated 4(SPATA4) Homo sapiens \| \| \| \|  \|  \|  \|  \| \| SPEG SPEG complex locus(SPEG) Homo sapiens \| \| \|  \|  \|  \|  \|  \| \| SPINK6 serine peptidase inhibitor, Kazal type 6(SPINK6) Homo sapiens \| \| \| \| \|  \|  \|  \| \| SPOCD1 SPOC domain containing 1(SPOCD1) Homo sapiens \| \| \| \|  \|  \|  \|  \| \| SPOCK1 SPARC/osteonectin, cwcv and kazal like domains proteoglycan 1(SPOCK1) Homo sapiens \| \| \| \| \| \| \|  \| \| SPOCK3 SPARC/osteonectin, cwcv and kazal like domains proteoglycan 3(SPOCK3) Homo sapiens \| \| \| \| \| \| \|  \| \| SPP1 secreted phosphoprotein 1(SPP1) Homo sapiens \| \| \|  \|  \|  \|  \|  \| \| SPRED1 sprouty related EVH1 domain containing 1(SPRED1) Homo sapiens \| \| \| \| \|  \|  \|  \| \| SPRED3 sprouty related EVH1 domain containing 3(SPRED3) Homo sapiens \| \| \| \| \|  \|  \|  \| \| SPRY1 sprouty RTK signaling antagonist 1(SPRY1) Homo sapiens \| \| \| \|  \|  \|  \|  \| \| SPRY2 sprouty RTK signaling antagonist 2(SPRY2) Homo sapiens \| \| \| \|  \|  \|  \|  \| \| SPRY4 sprouty RTK signaling antagonist 4(SPRY4) Homo sapiens \| \| \| \|  \|  \|  \|  \| \| SPRYD5 SPRY Domain Containing 5 or Tripartite Motif-Containing 51 (TRIM51) Homo Sapiens \| \| \| \| \| \| \|  \| \| SPTB spectrin beta, erythrocytic(SPTB) Homo sapiens \| \| \|  \|  \|  \|  \|  \| \| SPTLC3 serine palmitoyltransferase long chain base subunit 3(SPTLC3) Homo sapiens \| \| \| \| \| \|  \|  \| \| SSTR1 somatostatin receptor 1(SSTR1) Homo sapiens \| \| \|  \|  \|  \|  \|  \| \| ST3GAL1 ST3 beta-galactoside alpha-2,3-sialyltransferase 1(ST3GAL1) Homo sapiens \| \| \| \| \| \|  \|  \| \| ST6GAL1 ST6 beta-galactoside alpha-2,6-sialyltransferase 1(ST6GAL1) Homo sapiens \| \| \| \| \| \|  \|  \| \| ST6GALNAC4 ST6 N-acetylgalactosaminide alpha-2,6-sialyltransferase 4(ST6GALNAC4) Homo sapiens \| \| \| \| \| \| \|  \| \| STC1 stanniocalcin 1(STC1) Homo sapiens \| \|  \|  \|  \|  \|  \|  \| \| STEAP2 STEAP2 metalloreductase(STEAP2) Homo sapiens \| \| \|  \|  \|  \|  \|  \| \| STMN3 stathmin 3(STMN3) Homo sapiens \| \|  \|  \|  \|  \|  \|  \| \| SULT1C2 sulfotransferase family 1C member 2(SULT1C2) Homo sapiens \| \| \| \|  \|  \|  \|  \| \| SUMF1 sulfatase modifying factor 1(SUMF1) Homo sapiens \| \| \|  \|  \|  \|  \|  \| \| SUSD4 sushi domain containing 4(SUSD4) Homo sapiens \| \| \|  \|  \|  \|  \|  \| \| SYBU syntabulin(SYBU) Homo sapiens \| \|  \|  \|  \|  \|  \|  \| \| SYNC syncoilin, intermediate filament protein(SYNC) Homo sapiens \| \| \| \|  \|  \|  \|  \| \| SYNE2 spectrin repeat containing nuclear envelope protein 2(SYNE2) Homo sapiens \| \| \| \| \| \|  \|  \| \| SYT17 synaptotagmin 17(SYT17) Homo sapiens \| \|  \|  \|  \|  \|  \|  \| \| SYTL5 synaptotagmin like 5(SYTL5) Homo sapiens \| \| \|  \|  \|  \|  \|  \| \| TAGLN transgelin(TAGLN) Homo sapiens \| \|  \|  \|  \|  \|  \|  \| \| TAGLN2 transgelin 2(TAGLN2) Homo sapiens \| \|  \|  \|  \|  \|  \|  \| \| TAPBPL TAP binding protein like(TAPBPL) Homo sapiens \| \| \|  \|  \|  \|  \|  \| \| TBC1D2 TBC1 domain family member 2(TBC1D2) Homo sapiens \| \| \| \|  \|  \|  \|  \| \| TBX2 T-box 2(TBX2) Homo sapiens \|  \|  \|  \|  \|  \|  \|  \| \| TEC tec protein tyrosine kinase(TEC) Homo sapiens \| \| \|  \|  \|  \|  \|  \| \| TEC transient erythroblastopenia of childhood(TEC) Homo sapiens \| \| \| \|  \|  \|  \|  \| \| TGFA transforming growth factor alpha(TGFA) Homo sapiens \| \| \|  \|  \|  \|  \|  \| \| TGFB1 transforming growth factor beta 1(TGFB1) Homo sapiens \| \| \| \|  \|  \|  \|  \| \| TGFB2 transforming growth factor beta 2(TGFB2) Homo sapiens \| \| \| \|  \|  \|  \|  \| \| TGFBI transforming growth factor beta induced(TGFBI) Homo sapiens \| \| \| \|  \|  \|  \|  \| \| THBS3 thrombospondin 3(THBS3) Homo sapiens \| \|  \|  \|  \|  \|  \|  \| \| TIAM1 T-cell lymphoma invasion and metastasis 1(TIAM1) Homo sapiens \| \| \| \| \|  \|  \|  \| \| TIMP3 TIMP metallopeptidase inhibitor 3(TIMP3) Homo sapiens \| \| \| \|  \|  \|  \|  \| \| TLE3 transducin like enhancer of split 3(TLE3) Homo sapiens \| \| \| \|  \|  \|  \|  \| \| TLL1 tolloid like 1(TLL1) Homo sapiens \|  \|  \|  \|  \|  \|  \|  \| \| TLR3 toll like receptor 3(TLR3) Homo sapiens \| \|  \|  \|  \|  \|  \|  \| \| TMC6 transmembrane channel like 6(TMC6) Homo sapiens \| \| \|  \|  \|  \|  \|  \| \| TMEM130 transmembrane protein 130(TMEM130) Homo sapiens \| \| \| \|  \|  \|  \|  \| \| TMEM132A transmembrane protein 132A(TMEM132A) Homo sapiens \| \| \| \| \|  \|  \|  \| \| TMEM132D transmembrane protein 132D(TMEM132D) Homo sapiens \| \| \| \| \|  \|  \|  \| \| TMEM184A transmembrane protein 184A(TMEM184A) Homo sapiens \| \| \| \| \|  \|  \|  \| \| TMEM189-UBE2V1 TMEM189-UBE2V1 readthrough(TMEM189-UBE2V1) Homo sapiens \| \| \| \| \| \|  \|  \| \| TMEM2 transmembrane protein 2(TMEM2) Homo sapiens \| \| \|  \|  \|  \|  \|  \| \| TMEM229B transmembrane protein 229B(TMEM229B) Homo sapiens \| \| \| \| \|  \|  \|  \| \| TMEM238 transmembrane protein 238(TMEM238) Homo sapiens \| \| \| \|  \|  \|  \|  \| \| TMEM40 transmembrane protein 40(TMEM40) Homo sapiens \| \| \| \|  \|  \|  \|  \| \| TMEM56 transmembrane protein 56(TMEM56) Homo sapiens \| \| \| \|  \|  \|  \|  \| \| TMEM59L transmembrane protein 59 like(TMEM59L) Homo sapiens \| \| \| \|  \|  \|  \|  \| \| TMPRSS3 transmembrane protease, serine 3(TMPRSS3) Homo sapiens \| \| \| \| \|  \|  \|  \| \| TMSB4X thymosin beta 4, X-linked(TMSB4X) Homo sapiens \| \| \| \|  \|  \|  \|  \| \| TMTC2 transmembrane and tetratricopeptide repeat containing 2(TMTC2) Homo sapiens \| \| \| \| \| \|  \|  \| \| TMTC4 transmembrane and tetratricopeptide repeat containing 4(TMTC4) Homo sapiens \| \| \| \| \| \|  \|  \| \| TNC tenascin C(TNC) Homo sapiens \|  \|  \|  \|  \|  \|  \|  \| \| TNFRSF9 TNF receptor superfamily member 9(TNFRSF9) Homo sapiens \| \| \| \| \|  \|  \|  \| \| TNNT1 troponin T1, slow skeletal type(TNNT1) Homo sapiens \| \| \|  \|  \|  \|  \|  \| \| TNS1 tensin 1(TNS1) Homo sapiens \|  \|  \|  \|  \|  \|  \|  \| \| TOX thymocyte selection associated high mobility group box(TOX) Homo sapiens \| \| \| \| \|  \|  \|  \| \| TRANK1 tetratricopeptide repeat and ankyrin repeat containing 1(TRANK1) Homo sapiens \| \| \| \| \| \|  \|  \| \| TRIB2 tribbles pseudokinase 2(TRIB2) Homo sapiens \| \| \|  \|  \|  \|  \|  \| \| TRIM22 tripartite motif containing 22(TRIM22) Homo sapiens \| \| \|  \|  \|  \|  \|  \| \| TRIM29 tripartite motif containing 29(TRIM29) Homo sapiens \| \| \|  \|  \|  \|  \|  \| \| TRIM54 tripartite motif containing 54(TRIM54) Homo sapiens \| \| \|  \|  \|  \|  \|  \| \| TRIM58 tripartite motif containing 58(TRIM58) Homo sapiens \| \| \|  \|  \|  \|  \|  \| \| TRIM6 tripartite motif containing 6(TRIM6) Homo sapiens \| \| \|  \|  \|  \|  \|  \| \| TRIML2 tripartite motif family like 2(TRIML2) Homo sapiens \| \| \|  \|  \|  \|  \|  \| \| TRPC4 transient receptor potential cation channel subfamily C member 4(TRPC4) Homo sapiens \| \| \| \| \| \|  \|  \| \| TRPM3 transient receptor potential cation channel subfamily M member 3(TRPM3) Homo sapiens \| \| \| \| \| \| \|  \| \| TSC22D1 TSC22 domain family member 1(TSC22D1) Homo sapiens \| \| \| \|  \|  \|  \|  \| \| TSC22D3 TSC22 domain family member 3(TSC22D3) Homo sapiens \| \| \| \|  \|  \|  \|  \| \| TSPAN10 tetraspanin 10(TSPAN10) Homo sapiens \| \|  \|  \|  \|  \|  \|  \| \| TSPAN15 tetraspanin 15(TSPAN15) Homo sapiens \| \|  \|  \|  \|  \|  \|  \| \| TTC3P1 tetratricopeptide repeat domain 3 pseudogene 1(TTC3P1) Homo sapiens \| \| \| \| \|  \|  \|  \| \| TUBA4A tubulin alpha 4a(TUBA4A) Homo sapiens \| \| \|  \|  \|  \|  \|  \| \| TXK TXK tyrosine kinase(TXK) Homo sapiens \| \|  \|  \|  \|  \|  \|  \| \| TXNIP thioredoxin interacting protein(TXNIP) Homo sapiens \| \| \|  \|  \|  \|  \|  \| \| UBA7 ubiquitin like modifier activating enzyme 7(UBA7) Homo sapiens \| \| \| \| \|  \|  \|  \| \| UBE2QL1 ubiquitin conjugating enzyme E2 Q family like 1(UBE2QL1) Homo sapiens \| \| \| \| \| \|  \|  \| \| UCA1 urothelial cancer associated 1 (non-protein coding)(UCA1) Homo sapiens \| \| \| \| \|  \|  \|  \| \| UCN2 urocortin 2(UCN2) Homo sapiens \| \|  \|  \|  \|  \|  \|  \| \| UNC13D unc-13 homolog D(UNC13D) Homo sapiens \| \| \|  \|  \|  \|  \|  \| \| UPK1A uroplakin 1A(UPK1A) Homo sapiens \| \|  \|  \|  \|  \|  \|  \| \| UPK1A-AS1 UPK1A antisense RNA 1(UPK1A-AS1) Homo sapiens \| \| \| \|  \|  \|  \|  \| \| USP53 ubiquitin specific peptidase 53(USP53) Homo sapiens \| \| \|  \|  \|  \|  \|  \| \| VAMP5 vesicle associated membrane protein 5(VAMP5) Homo sapiens \| \| \| \|  \|  \|  \|  \| \| VAMP8 vesicle associated membrane protein 8(VAMP8) Homo sapiens \| \| \| \|  \|  \|  \|  \| \| VGLL2 vestigial like family member 2(VGLL2) Homo sapiens \| \| \|  \|  \|  \|  \|  \| \| VGLL3 vestigial like family member 3(VGLL3) Homo sapiens \| \| \|  \|  \|  \|  \|  \| \| VLDLR very low density lipoprotein receptor(VLDLR) Homo sapiens \| \| \| \|  \|  \|  \|  \| \| WDR69 WD Repeat Domain 69 or Dynein Assembly Factor With WD Repeats 1 (DAW1) Homo Sapiens \| \| \| \| \| \| \| \| \| WFDC3 WAP four-disulfide core domain 3(WFDC3) Homo sapiens \| \| \| \|  \|  \|  \|  \| \| WFS1 wolframin ER transmembrane glycoprotein(WFS1) Homo sapiens \| \| \| \|  \|  \|  \|  \| \| WISP2 WNT1 inducible signaling pathway protein 2(WISP2) Homo sapiens \| \| \| \| \|  \|  \|  \| \| WISP3 WNT1 inducible signaling pathway protein 3(WISP3) Homo sapiens \| \| \| \| \|  \|  \|  \| \| WNK4 WNK lysine deficient protein kinase 4(WNK4) Homo sapiens \| \| \| \|  \|  \|  \|  \| \| WNT5B Wnt family member 5B(WNT5B) Homo sapiens \| \| \|  \|  \|  \|  \|  \| \| WNT7A Wnt family member 7A(WNT7A) Homo sapiens \| \| \|  \|  \|  \|  \|  \| \| WNT7B Wnt family member 7B(WNT7B) Homo sapiens \| \| \|  \|  \|  \|  \|  \| \| WNT9A Wnt family member 9A(WNT9A) Homo sapiens \| \| \|  \|  \|  \|  \|  \| \| WWC1 WW and C2 domain containing 1(WWC1) Homo sapiens \| \| \| \|  \|  \|  \|  \| \| WWTR1 WW domain containing transcription regulator 1(WWTR1) Homo sapiens \| \| \| \| \|  \|  \|  \| \| XG Xg blood group(XG) Homo sapiens \| \|  \|  \|  \|  \|  \|  \| \| ZC4H2 zinc finger C4H2-type containing(ZC4H2) Homo sapiens \| \| \|  \|  \|  \|  \|  \| \| ZCCHC24 zinc finger CCHC-type containing 24(ZCCHC24) Homo sapiens \| \| \| \|  \|  \|  \|  \| \| ZCCHC5 zinc finger CCHC-type containing 5(ZCCHC5) Homo sapiens \| \| \| \|  \|  \|  \|  \| \| ZDHHC2 zinc finger DHHC-type containing 2(ZDHHC2) Homo sapiens \| \| \| \|  \|  \|  \|  \| \| ZNF165 zinc finger protein 165(ZNF165) Homo sapiens \| \| \|  \|  \|  \|  \|  \| \| ZNF323 zinc finger protein 323(ZNF323) Homo sapiens \| \| \|  \|  \|  \|  \|  \| \| ZNF488 zinc finger protein 488(ZNF488) Homo sapiens \| \| \|  \|  \|  \|  \|  \| \| ZNF610 zinc finger protein 610(ZNF610) Homo sapiens \| \| \|  \|  \|  \|  \|  \| \| ZNF676 zinc finger protein 676(ZNF676) Homo sapiens \| \| \|  \|  \|  \|  \|  \| \| ZNF703 zinc finger protein 703(ZNF703) Homo sapiens \| \| \|  \|  \|  \|  \|  \| \| ZNF716 zinc finger protein 716(ZNF716) Homo sapiens \| \| \|  \|  \|  \|  \|  \| \| ZNF804A zinc finger protein 804A(ZNF804A) Homo sapiens \| \| \|  \|  \|  \|  \|  \| \| ZSCAN18 zinc finger and SCAN domain containing 18(ZSCAN18) Homo sapiens \| \| \| \| \|  \|  \|  \| | | | | | | | |  |  |  |  |  |  |  |  |
